# The Chemokine System as a Key Regulator of Pulmonary Fibrosis: Converging Pathways in Human Idiopathic Pulmonary Fibrosis (IPF) and the Bleomycin-Induced Lung Fibrosis Model in Mice

**DOI:** 10.3390/cells13242058

**Published:** 2024-12-12

**Authors:** Remo Castro Russo, Bernhard Ryffel

**Affiliations:** 1 Laboratory of Pulmonary Immunology and Mechanics, Department of Physiology and Biophysics, Institute of Biological Sciences, Universidade Federal de Minas Gerais-UFMG, Belo Horizonte 31270-901, MG, Brazil; 2 Laboratory of Immuno-Neuro Modulation (INEM), UMR7355 Centre National de la Recherche Scientifique (CNRS), University of Orleans, 45071 Orleans, France

**Keywords:** chemokine, mucosal immunology, interstitial lung disease, idiopathic pulmonary fibrosis, therapeutic targets

## Abstract

Idiopathic pulmonary fibrosis (IPF) is a chronic and lethal interstitial lung disease (ILD) of unknown origin, characterized by limited treatment efficacy and a fibroproliferative nature. It is marked by excessive extracellular matrix deposition in the pulmonary parenchyma, leading to progressive lung volume decline and impaired gas exchange. The chemokine system, a network of proteins involved in cellular communication with diverse biological functions, plays a crucial role in various respiratory diseases. Chemokine receptors trigger the activation, proliferation, and migration of lung-resident cells, including pneumocytes, endothelial cells, alveolar macrophages, and fibroblasts. Around 50 chemokines can potentially interact with 20 receptors, expressed by both leukocytes and non-leukocytes such as tissue parenchyma cells, contributing to processes such as leukocyte mobilization from the bone marrow, recirculation through lymphoid organs, and tissue influx during inflammation or immune response. This narrative review explores the complexity of the chemokine system in the context of IPF and the bleomycin-induced lung fibrosis mouse model. The goal is to identify specific chemokines and receptors as potential therapeutic targets. Recent progress in understanding the role of the chemokine system during IPF, using experimental models and molecular diagnosis, underscores the complex nature of this system in the context of the disease. Despite advances in experimental models and molecular diagnostics, discovering an effective therapy for IPF remains a significant challenge in both medicine and pharmacology. This work delves into microarray results from lung samples of IPF patients and murine samples at different stages of bleomycin-induced pulmonary fibrosis. By discussing common pathways identified in both IPF and the experimental model, we aim to shed light on potential targets for therapeutic intervention. Dysregulation caused by abnormal chemokine levels observed in IPF lungs may activate multiple targets, suggesting that chemokine signaling plays a central role in maintaining or perpetuating lung fibrogenesis. The highlighted chemokine axes (CCL8-CCR2, CCL19/CCL21-CCR7, CXCL9-CXCR3, CCL3/CCL4/CCL5-CCR5, and CCL20-CCR6) present promising opportunities for advancing IPF treatment research and uncovering new pharmacological targets within the chemokine system.

## 1. Introduction

Inflammation is a dynamic response to tissue trauma, injury, or infections, with the goal of restoring tissue function and homeostasis [1,2,3]. Mediators of inflammation guide the immune response either locally or systemically, coordinating the components of innate and adaptive immunity [4]. Chemokines, initially classified as members of the cytokine family and also known as “chemoattractant cytokines” [5], play a pivotal role in inflammatory processes. They direct the recruitment and activation of leukocytes into inflammatory sites [6,7,8,9,10] and participate in angiogenesis [11], resolution of inflammation, and tissue repair [1,8,12]. Tissue repair is a protective response following injury, that replaces acute inflammation by regenerative processes, aiming at restoring tissue homeostasis [4,13]. However, fibrosis resulting from chronic injury can lead to tissue degeneration and dysfunction. Functional repair, which involves replacing damaged tissues with connective tissue, may result in defective healing characterized by abnormal architecture and excessive extracellular matrix (ECM) deposition [12]. This review examines the role of chemokines in idiopathic pulmonary fibrosis (IPF) and bleomycin-induced lung fibrosis, integrating insights from both patient and animal studies. It also discusses potential therapeutic strategies targeting chemokine pathways and highlights the limitations of current approaches.

## 2. The Complexity and Physiological Importance of the Chemokine System

Chemokines (8–12 kDa) regulate leukocyte biology by controlling communication, recruitment, and activation in both basal and inflammatory states, impacting immune responses from initiation to resolution [5,9,14]. They also affect non-leukocyte cell types, including epithelial, endothelial, fibroblast, and smooth muscle cells [14,15]. Chemokines and their receptors influence various physiological functions, from chemotaxis to tissue remodeling, contributing to pathophysiological processes like chronic inflammation and tumorigenesis [5,9,10,14,15,16,17]. Notably, receptors such as CCR5 and CXCR4 also act as co-receptors for HIV [18]. These findings underscore the diverse role of chemokines in maintaining homeostasis and influencing tissue pathology [8,9,11,15,17], highlighting their significance in both normal and pathological conditions.

The chemokine system, found in all vertebrates and mammals, comprises approximately 50 ligands in humans. These ligands, either constitutively present or induced by stimuli, interact with about 20 receptors. These receptors, with seven transmembrane domains, belong to the class A of G protein-coupled receptors (GPCRs) in the Rhodopsin-like family [6,10,16]. Despite their promiscuous binding, chemokines exhibit unique signaling pathways and activities. However, functional redundancy exists as most receptors interact with multiple ligands, and conversely, most chemokines interact with more than one receptor [10,14,16]. Chemokines, which are structurally related, are classified based on the positioning of conserved cysteine residues. CC chemokines (β-chemokines) feature adjacent first two cysteines, while CXC (α-chemokines) exhibit a single amino acid separation, leading to sub-groups CXC-ELR- and CXC-ELR+ with the ELR motif [10,16]. CX3C has three amino acids between the first two cysteines, and C chemokines lack the first and third cysteines [7,8,9,10]. This system, through diverse chemokine receptor expression in various cell types, influences leukocyte migration and activation in the presence of chemokine gradients [6,10,15,16]. Currently, the chemokine system includes 28 CC chemokines (CCL1-28), 17 CXC chemokines (CXCL1-17), 2 C chemokines (XCL1-2), and 1 CX3C chemokine (CX3CL1). These chemokines engage a wide range of receptors: 10 CCR receptors (CCR1-10), 6 CXCR receptors (CXCR1-6), 1 XCR receptor (XCR1), and 1 CX3CR receptor (CX3CR1) [10,15,16]. This complex network highlights the diversity and specificity of the chemokine system (Table 1), highlighting its important role in cellular communication and immune regulation.

Chemokines, binding to sulfated heparan, heparin, and glycosaminoglycans in the extracellular matrix, serve as docking sites for haptotaxis, regulating the soluble chemokine gradient and chemotaxis [10,16,21,22,23]. In inflammation, various proteases (serine, metallo-, dipeptidyl peptidases) cleave chemokines, creating a solubility-based gradient that alters chemokine states and receptor activity. Most chemokines are produced and secreted, with the exception of CX3CL1 and CXCL16, transmembrane molecules requiring proteolytic cleavage for a soluble chemotactic gradient [10,16,24]. Exhibiting dual activity, chemokines act as natural receptor antagonists, influencing specific immune pathways. For instance, certain CXCR receptor ligands can function as antagonists of the CCR receptor [10,16,25,26,27,28]. This dual role enables chemokines to bind as agonists to their receptors while simultaneously exhibiting inhibitory effects on other receptors.

**Figure 1:**
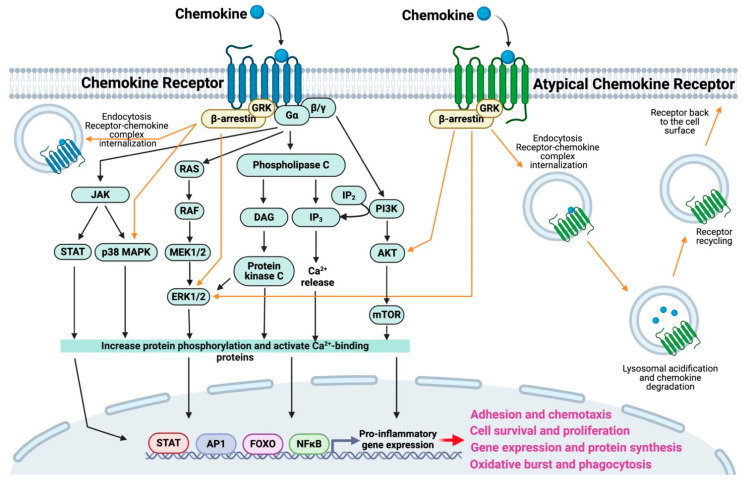
Chemokine receptor and atypical chemokine receptor (ACKR) activation and signaling. Chemokines bind to classical chemokine receptors, which are G protein-coupled receptors (GPCRs) expressed on cell surfaces, sensing the presence of chemokines, and initiating critical intracellular signaling cascades during inflammation. Upon activation, GPCRs dissociate G protein subunits Gα and Gβγ, triggering key pathways of Phospholipase C (PLC) and Phosphoinositide 3-Kinase (PI3K). PLC cleaves phosphatidylinositol 4,5-bisphosphate (PIP2) into Inositol triphosphate (IP3), which promotes calcium release from intracellular stores; and Diacylglycerol (DAG) activates protein kinase C (PKC), influencing migration and gene expression. PI3K activation by chemokine receptor signaling, converts PIP2 to phosphatidylinositol 3,4,5-trisphosphate (PIP3), activating the PI3K/AKT pathway, crucial for survival and motility. Additionally, GPCRs engage the JAK/STAT and Ras/Raf/ERK pathways, driving proliferation, differentiation, and adhesion. Phosphorylation by GPCR kinases (GRKs) recruits β-arrestin, leading to receptor internalization and activation of ERK and MAPK pathways. These cascades activate transcription factors such as STAT, FOXO, AP-1, and NF-κB, promoting the expression of inflammatory genes. This orchestrates cellular activation, adhesion, and migration, central to inflammation and immune responses. Atypical chemokine receptors (ACKRs) modulate chemokine activity by binding chemokines and functioning as scavengers. Upon ligand binding, ACKRs undergo phosphorylation by GPCR kinases (GRKs), leading to β-arrestin recruitment. This process facilitates the internalization of chemokines and their subsequent lysosomal degradation, effectively reducing extracellular chemokine levels. Following ligand degradation, ACKRs are recycled back to the cell surface through intracellular trafficking mechanisms, enabling them to continue their regulatory functions. Additionally, β-arrestin recruitment activates MAPK-related-signaling pathways, particularly ERK1/2 and AKT, which are critical for promoting cell survival and proliferation. By scavenging chemokines and preventing their interaction with classical chemokine receptors, ACKRs play a key role in fine-tuning extracellular chemokine concentrations. This mechanism diminishes chemokine signaling through traditional receptors, thereby regulating immune responses and preventing excessive inflammation. Red arrows illustrate the cellular processes initiated by signaling pathways activated via chemokine receptors. Black arrows denote the signaling pathways induced by the G protein subunits Gα and Gβγ. Orange arrows indicate the signaling pathways activated through β-arrestin in chemokine receptors.

Chemokine receptors are located in lipid rafts floating on the cell membrane, and function as signal sensors, relaying extracellular chemical messages. Facilitated by cholesterol micelles, receptor oligomerization leads to the formation of homodimers and heterodimers, inducing structural and signaling changes [10,16]. This process unlocks functionalities like trans-activation, co-internalization, and cross-desensitization. Higher concentrations of naturally occurring chemokine oligomers enhance their activity [6,29]. Chemokine receptors interact by binding to chemokines [7], triggering a conformational change that leads to receptor endocytosis and activation of intracellular signals (Figure 1) [10,16]. Upon agonist binding, the receptor proceeds into a pre-activation state coupling with the heterotrimeric G protein, where the exchange of GDP and GTP in G protein α subunit leads to G protein dissociation and mediates the G protein-signaling pathway. Together, the signaling of Gα and Gβ/γ initiates toward increased concentrations of the chemokines, activating Adenylate Cyclase, Phospholipase-Cβ, PI3K, and Small GTPases. This modification in the cell activation state is characterized by elevated cyclic AMP, IP3 phosphorylation, and intracellular Ca^2+^ levels [9,10,16,30,31]. The phosphorylation of the receptor C-terminal tail by GRK binding promotes β-arrestin recruitment and signaling by chemokine receptor endocytosis. Chemokine receptor activation enhances many cellular processes, such as increasing cell survival, cellular motion by actin filament polymerization and cell polarization, and also improving the phagocytic capacity. It induces NF-κB translocation of pro-inflammatory gene transcription (Figure 1), with increased chemokine production, and de novo expression of their receptors [6,7,8,31]. The interaction and signaling of the chemokine-receptor play crucial roles in modifying cellular machinery, recycling receptors, and degrading chemokines. This regulation of endocytosis is pivotal for controlling membrane receptor levels, influencing cellular activity, and acting as a natural scavenger in pathological and homeostatic conditions [8,10,16,32,33].

The chemokine system includes atypical chemokine receptors (ACKRs), categorized as scavengers (Figure 1), which exhibit promiscuous binding to agonists without initiating canonical intracellular activation signals [8,16,20,32,33,34,35]. Structurally similar to G protein-coupled receptors (GPCRs), ACKRs are naturally uncoupled from canonical GPCR-signaling machinery due to alterations in the DRYLAIV motif [8,16,32,33,34,35]. Unlike typical chemokine receptors, ACKRs function as decoys, reducing chemokine levels by sequestering and degrading ligands. This strategy competes with GPCR-signaling receptors, providing post-transcriptional chemokine level control, and acting as a negative regulator of inflammation [16,36,37,38]. Currently, five ACKRs with scavenging functions are known to bind chemokines with high affinity (Table 1). These receptors internalize chemokines through endocytosis, followed by their degradation in lysosomes (Figure 1). After this process, ACKRs are recycled back to the cell surface, enabling them to continue regulating chemokine availability and preventing excessive chemokine signaling. This recycling mechanism ensures that ACKRs maintain their role in modulating immune responses by controlling extracellular chemokine concentrations [10,16,20,34,35]. ACKR1/DARC exhibits broad promiscuity, binding both CC and CXC chemokines. Located in erythrocytes, ACKR1 facilitates chemokine transport from the sub-luminal face to the luminal surface of vascular endothelium, supporting the leukocyte trans-endothelial migration [16,20,34,35,36,39]. ACKR2/D6 selectively binds to inflammatory chemokines, acting as a gatekeeper on lymphatic endothelial cells to regulate leukocyte entry into lymph nodes and the placenta, thereby inhibiting inflammation during fetal development. ACKR2 is also present in barrier tissues and organs like the liver, spleen, thyroid, and on leukocytes at inflammation sites in the skin and lungs [8,16,35,36,37,40,41,42,43,44]. In contrast, ACKR3/CXCR7 and ACKR4/CCX-CKR function as scavenger receptors for CXC and CC chemokines, respectively, with homeostatic properties [10,16,34,35,45]. GPR182 has recently been identified as ACKR5, expressed in the microvascular and lymphatic endothelial cells of most organs and is involved in hematopoietic stem cell homeostasis [46,47], but requires further investigation to clarify its roles [16,20,34,35]. Collectively, ACKRs regulate chemokine levels precisely, influencing tissue homeostasis and immune responses.

The complexity of the chemokine system, which is crucial in modulating immune functions during inflammation and infection, is confronted with challenges from pathogen evasion mechanisms. These challenges include pathogen-encoded chemokines and decoy proteins such as soluble receptors, chemokine-binding proteins (CKBPs), and glycosaminoglycan antagonists [10,16]. Pathogens, including viruses (e.g., herpesvirus, poxvirus), protozoa (*Plasmodium* sp., *Toxoplasma gondii*), helminths (*Schistosoma mansoni*), and blood ectoparasites like ticks (*Rhipicephalus sanguineus*), employ these strategies [8,48,49,50]. A new class of molecules, “chemokine-like function” (CLF), which is distinct from chemokines, can activate and signal through chemokine receptors [10,16,51]. Non-cognate endogenous ligands such as β-defensins (binding to CCR6), Macrophage Migration Inhibitory Factor (MIF) recognized by CXCR2 and CXCR4, and the Pro-Gly-Pro tripeptide (PGP), released during collagen degradation by metalloproteinases (MMPs), contribute to the increased complexity of the chemokine system regulation [10,16,52,53,54].

### Therapeutic Targeting of Chemokines and Their Receptors

In the last three decades since the discovery of the first chemokine [10,16], research has revealed its diverse roles in inflammation, autoimmune diseases, allergies, infectious diseases, cancer, and AIDS [10,16,18]. Pharmacological studies using neutralizing antibodies or inhibitors targeting chemokine receptor signaling demonstrate the therapeutic potential in inflammatory pathologies [10,16]. Despite these promising avenues, the nonspecific interaction between chemokines and receptors poses challenges for selective drug development [10,16,55,56]. Various compounds that modify chemokine activity have been introduced to disrupt their interaction with receptors [10,16].

Anti-chemokine antibodies and chemokine-binding proteins (CKBPs) interact with chemokines, modifying their activity on chemokine receptors [10,16,56]. Disruption of chemokine activity includes the use of anti-receptor antibodies, small-molecule orthosteric antagonists at the binding site, or allosteric antagonists that induce conformational changes [10,16,55,56]. Modified chemokines and inhibitors compete for anchoring sites on matrix glycosaminoglycans, thereby hindering the formation of a chemotactic gradient [10,16,56].

Recent focus has been on inhibiting intracellular signaling pathways (e.g., PI3K, mTOR, MAP kinases) activated by chemokine receptors for drug development [10,30]. Blocking signaling from the chemokine-receptor complex negatively regulates biological activity in inflammatory or proliferating cells, affecting shared pathways among multiple receptors [10,30]. Targeting chemokines and receptors is crucial for therapeutic intervention in inflammatory and autoimmune diseases to inhibit pathological migration.

## 3. Significance of Chemokines in Pulmonary Disorders and Idiopathic Pulmonary Fibrosis (IPF)

Chemokines and their receptors play distinct roles in various tissues and disease contexts [57], forming a complex signaling network that regulates functions in different leukocyte types and cell populations [58,59]. In pulmonary pathophysiology, chemokines are crucial for processes such as acute lung injury (ALI) and inflammation [60,61,62,63,64], inducible bronchus-associated lymphoid tissue (iBALT) formation [65], pulmonary arterial hypertension [66], asthma [67], chronic obstructive pulmonary disease (COPD) [68], tumorigenesis [69], and pulmonary fibrosis [70,71,72]. This discussion focuses on their roles in idiopathic pulmonary fibrosis (IPF), emphasizing involvement in lung inflammation and tissue repair processes.

Pulmonary fibrosis, the final stage of interstitial lung disease (ILD), encompasses a group of lung disorders with shared clinical features and varying morbidity based on pathological, physiological, and radiographic changes [73,74]. IPF, a chronic and life-threatening form within ILDs, has unknown origins and limited treatment options. It is characterized by fibroproliferation and excessive extracellular matrix deposition in the pulmonary parenchyma [74,75,76,77,78]. Clinical manifestations include worsening dyspnea, progressive loss of lung volumes [79,80,81], and abnormal gas exchange, which can lead to respiratory collapse [75,76,77,78]. IPF affects circa 3 million people worldwide, with a substantial increase in incidence in older patients [82]. With approximately 30 cases per 100,000 people and around 34,000 new cases annually in the United States [74,82,83], IPF has a high mortality rate due to a gradual decline in lung function, resulting in respiratory failure, with an average survival of 3 to 5 years after diagnosis [74,78,79,82].

Research into the origin of IPF extensively investigates the triggering phenomena, but its ontogenesis remains controversial and poorly understood [84,85]. Unraveling the cellular and molecular events associated with IPF’s characteristic histopathological findings continues to be elusive despite extensive study. Various hypotheses suggest disrupted cross-talk between epithelial cells and fibroblasts or an inflammatory route leading to fibrosis as a consequence of prior alveolitis [84,85,86,87,88,89,90]. Pulmonary fibrosis frequently arises after chronic inflammatory lung damage, where improper tissue repair may lead to excessive fibrogenesis [74,87,90,91,92]. Imbalances in catabolic mechanisms and tissue regeneration functions are considered potential causes, resulting in excessive healing following prior chronic inflammation [91]. Clinical data also reveal a strong correlation between pulmonary fibrosis and inflammatory mediators released by leukocytes and resident cells [74,91,93,94].

IPF, influenced by both genetic and environmental factors, is a chronic inflammatory disorder that progresses to established fibrosis, characterized by repetitive local tissue damage and degeneration of alveolar epithelium [74,78,90]. Ongoing tissue stress disrupts communication between epithelial cells and fibroblasts, leading to myofibroblast matrix production and significant extracellular matrix accumulation, resulting in interstitial lung remodeling [74,90]. Evidence suggests that pulmonary fibrosis may develop through an inflammatory route, arising from prior alveolitis and excessive scarring [74,90,91,94,95]. Although corticosteroids could serve as an immunosuppressive strategy [75,96,97], their therapeutic application is limited due to adverse effects. Presently, the therapy for IPF is based on antifibrotics. Although Pirfenidone and Nintedanib are currently approved for treating idiopathic PF (IPF), patients still have high mortality rates and a median survival duration of only 3–5 years [98,99,100,101]. Despite the fact that FDA-approved drugs Pirfenidone and Nintedanib consistently slow disease progression, lung transplantation remains the sole curative treatment for IPF [75,96,97].

## 4. Exploring the Chemokine System in Pulmonary Fibrosis: Literature Insights

Our understanding of IPF has evolved from analyzing human samples and fibrosis animal models, which have elucidated pathways in pulmonary fibrosis [91,96,102,103]. In the past decade, genetic and transcriptomic studies have significantly enhanced insights into molecular and cellular changes in the human lung, thereby influencing IPF prognosis and therapeutics [74,97,102,104]. Although IPF’s pathophysiology is not yet fully understood, pivotal insights from animal models, which emphasize the crucial role of chemokines, have been instrumental in unraveling the disease’s molecular basis.

Bleomycin administration in mouse models is essential for studying pulmonary fibrogenesis, as it mirrors human processes and significantly impacts pre-clinical studies for developing IPF therapeutic drugs like Pirfenidone and Nintedanib [103,104,105]. Originally identified as an antineoplastic glycopeptide antibiotic from *Streptomyces verticillus*, bleomycin induces nonspecific pneumonia that progresses to pulmonary fibrosis, with 46% of cases advancing to fibrosis and some resulting in fatal outcomes [106]. This experimental induction of pulmonary fibrosis in rodents closely replicates cellular and biochemical changes in patients [107], providing a systematic model for studying pathogenesis [108,109,110]. Bleomycin causes tissue damage via oxidative stress, leading to DNA and RNA breaks, which ultimately result in cell necrosis and apoptosis [106,110].

Following this initial damage, lung inflammation is triggered and becomes self-sustaining, progressing to chronic inflammation with excessive repair and fibrosis [106,110]. Subsequent to the pulmonary tissue injury caused by bleomycin, which particularly affects epithelial, endothelial cells, and resident macrophages, various damage-associated molecular patterns (DAMPs) are released. These include uric acid, which activates NALP3 inflammasome, TLR2 and TLR4, MyD88, and IL-1R1 pathways [111,112]. Additionally, ATP signaling through the P2X7 receptor [113] induces IL-1β, B-cell activating factor (BAFF), IL-17A, and IL-23, which optimize inflammation [112,114,115]. The alarmin pathway IL-33/ST2 is also activated [116], collaborating with the source of chemokines, growth factors (FGF, EGF, VEGF), and metalloproteinases (MMP9, MMP12) to orchestrate subsequent inflammatory responses, chronic disease, and fibrosis [12,91,117,118,119,120,121]. These mediators promote the formation of fibroblast and myofibroblast *foci* by stimulating the proliferation of resident mesenchymal cells, recruiting circulating fibrocytes, and stimulating epithelial-to-mesenchymal transition in IPF [91,119,121,122].

**Figure 2:**
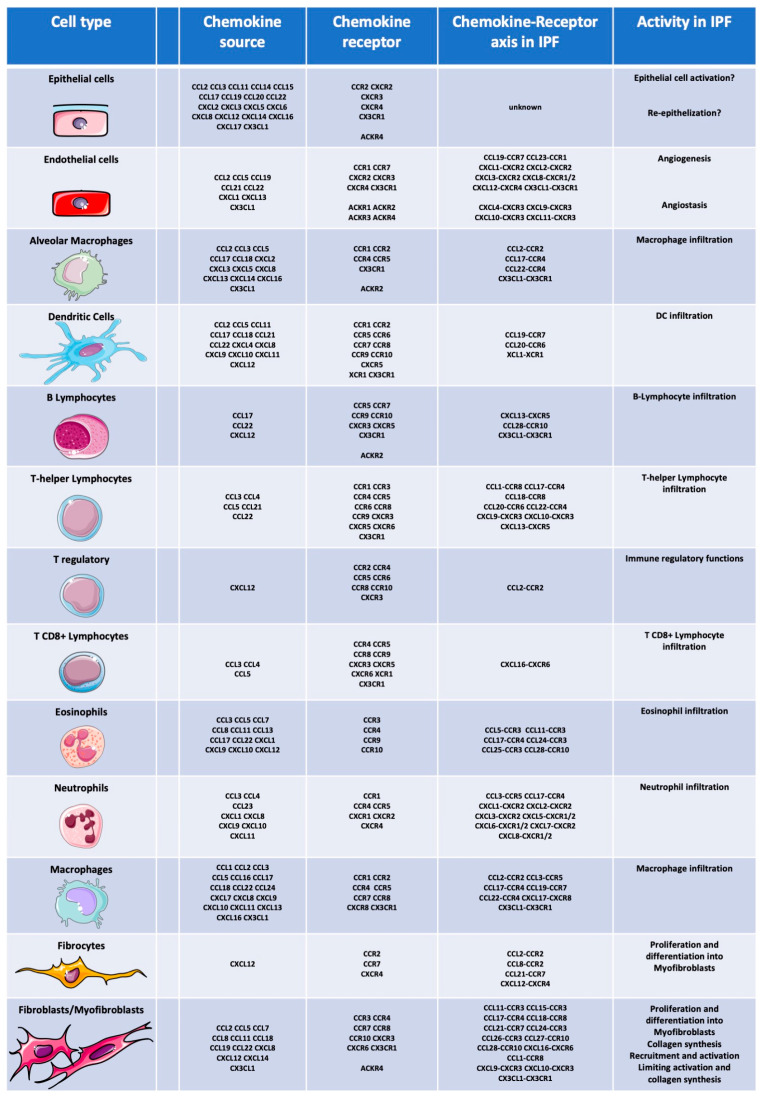
Cell types, chemokine and chemokine receptor expression, chemokine-receptor axis, and activities in idiopathic pulmonary fibrosis. The complexity of the chemokine system and its functions are illustrated based on the reviewed literature.

Chemokines and glycosaminoglycans play a pivotal role in IPF, affecting cell influx, angiogenesis, macrophage activation, and increased collagen production, all of which are implicated in IPF development [12,123]. As summarized in Figure 2, chemokines are produced by resident lung cells such as epithelial cells, fibroblasts and leukocytes (inducers) during lung fibrogenesis, and activation of their receptors on target cells such as fibroblasts and myofibroblasts (responders) which trigger diverse cellular phenomena including collagen and cytokine secretion, involved in fibrosis progression. Thus, understanding these mechanisms is crucial for developing effective pharmacological strategies. Recent insights from genetic and transcriptomic studies have enriched our understanding of molecular pathways in pulmonary fibrogenesis, with a focus on emerging cellular and molecular mechanisms involving the chemokine system in IPF onset and progression. The following section will explore the specific role of chemokines in IPF.

### 4.1. CC Chemokines in the Context of Pulmonary Fibrosis

CCL1: CCL1 (I-309) binds to CCR8, which is associated with Th2 immune response [10,16]. Research on its role in pulmonary fibrosis is limited. Elevated CCL1 levels in mouse models, primarily derived from alveolar macrophages and CD4+ T cells, were associated with reduced pathology when Ccl1 was deleted [124]. In vitro, deletion of CCR8 in fibroblasts restricted migration in response to CCL1, and blocked CCL1 improved fibrosis [124]. Antibody blockade of CCL1 improved PF pathology, supporting the therapeutic potential of targeting the CCL1-CCR8 axis in fibrosis [124]. Alveolar macrophages exposed to TGF-β in vitro underwent myofibroblast transdifferentiation with the involvement of CCR8 [125]. Targeting CCR8 could represent a novel therapeutic strategy for pulmonary fibrosis. CCL1 is implicated in T-cell recruitment in asthmatic lungs, dependent on CCR8 [126]. In mice, hMSC administration exacerbated lung fibrosis through persistent Ccl1 up-regulation [127]. Deletion of the CCL1 receptor Ccr8 in fibroblasts limited migration but not activation. However, CCL1 binds to AMFR, triggering ubiquitination of the ERK inhibitor Spry1, which activates Ras-mediated profibrotic protein synthesis [124]. Future studies may elucidate the role of the CCL1-CCR8 axis in pulmonary fibrosis.

CCL2: CCL2 (MCP-1/JE) binds to the CCR2 receptor and is associated with the innate immune response [10,16]. In the context of IPF, the role of CCL2-CCR2 is well characterized. CCL2 expression in IPF is identified in pulmonary epithelial cells, macrophages, vascular endothelial, and smooth muscle cells [128]. Lung epithelial cells from IPF patients strongly express CCL2 mRNA and protein compared to healthy samples, which sustains macrophage recruitment and lung infiltration in the pathological state [129,130]. Elevated CCL2 levels in bronchoalveolar lavage (BAL) correlate with low DLCO and arterial oxygen tension in IPF [131]. Increased CCL2 in BAL and serum may predict poor outcomes for IPF patients and aid in distinguishing IPF from other types of ILD [132,133]. In patients with acute exacerbation of IPF (AEIPF), reduced FVC and DLCO are observed, and CCL2 may impact overall survival, associated with macrophage activation [134]. In IPF lungs, certain monocytes express CD64hi and CCL2-expressing transitional macrophages that sustain CCL2 levels, attracting more monocytes into the lungs [135]. Activated IPF fibroblasts display the highest CCL2 production and contractility [136]. Circulating human fibrocytes expressing CCR2 demonstrate increased proliferation, differentiation into myofibroblasts, and a chemotactic response upon stimulation with CCL2 [137]. Isolated lung fibrocytes express CCR2, migrate in the presence of CCL2, and secrete collagen when stimulated by CCL2, suggesting that CCR2 regulates respiratory injury and fibrosis [138]. Tracking CCR2+ monocytes and macrophages in bleomycin- or radiation-induced fibrosis in mice and human samples of subjects with IPF (Clinical trial NCT03492762) indicates that PET uptake in the lung correlates with CCR2+ cell infiltration associated with fibrosis in mice [139]. In lung tissues of IPF patients, CCR2+ cells are concentrated in perifibrotic regions of fibrotic zones [139]. Inhibiting fibrosis using IL-1β blockade or antifibrotic pirfenidone reduces CCR2+ macrophage accumulation in mouse lungs. IPF samples contain significantly more CCL2 and M-CSF than BALF from healthy volunteers, and Ccl2^-/-^ mice are protected from bleomycin-induced pulmonary fibrosis, suggesting that M-CSF contributes to the pathogenesis of IPF through CCL2 production and activation of mononuclear phagocytes [140]. CCL2 and Proteinase-Activated Receptor-1 (PAR1) are expressed and up-regulated on the activated epithelium from IPF samples [141]. PAR1 activation on lung epithelial cells may enhance local CCL2 release in pulmonary fibrosis. CCL2-CCR2 signaling is pivotal in bleomycin-induced pulmonary fibrosis, influencing fibrogenic cytokine expression, fibroblast-to-myofibroblast differentiation, and TGF-β responsiveness [142]. Ccr2^-/-^ mice show reduced pulmonary fibrosis in response to bleomycin [142,143,144], with decreased myofibroblast differentiation [142], regulated macrophage infiltration and macrophage-derived MMP2 and MMP9 production [143], and lower fibrocyte mobilization [138]. Pirfenidone attenuates fibrocyte mobilization in bleomycin-induced pulmonary fibrosis in mice by reducing Ccl2 production in vivo and fibrocyte migration in vitro [145]. Gamma-herpesvirus infection up-regulates Ccl2 production and exacerbates pulmonary fibrosis in mice, with increased fibrocyte recruitment into the lungs in wild-type but abrogated in Ccr2^-/-^ mice [146]. Moreover, IL-10 induces fibrosis by fibrocyte recruitment and M2 macrophage activation dependent on the Ccl2/Ccr2 axis, with the abrogation of pulmonary fibrosis by treatment with anti-Ccl2 neutralizing antibodies in mice [147]. Mini-BAL and peripheral blood samples from chronic heart failure (CHF) exhibit increased CCL2 levels in BAL, along with M2 macrophages, IL-10, and TGF-β [148], contributing to pulmonary fibrotic remodeling and increased dyspnea severity. Patients with IPF show higher percentages of CCR2+ CD4+ T cells expressing Foxp3+ CD25+ within bronchoalveolar lavage fluid [149], suggesting immune regulatory functions of these leukocytes that could attenuate lung inflammation and fibrosis. The Carlumab, a human monoclonal antibody that specifically binds and neutralizes profibrotic activities of human CCL2, was tested in phase 2 of a clinical trial in IPF [150]. IPF patients treated with Carlumab showed no treatment benefit compared to the placebo, based on the primary endpoint of percentage change in FVC. All groups experienced FVC decline over time, with a greater reduction in the Carlumab-treated groups. Secondary analyses revealed no advantage in time to disease progression, absolute FVC change, or relative DLCO change compared to the placebo [150]. The most common adverse events (>20%) in Carlumab-treated patients were cough, fatigue, upper respiratory tract infections, and dyspnea [150].

CCL3: CCL3 (MIP-1α) binds to CCR1 and CCR5 receptors, influencing T-cell and monocyte/macrophage trafficking [10,16]. In IPF, CCL3 is found in BAL fluid [131], and expressed by alveolar and interstitial macrophages, interstitial fibroblasts [151] and BAL leukocytes in IPF patients [152]. Elevated CCL3 levels in BAL fluid correlate with increased neutrophil and eosinophil numbers in IPF patients [131], though this correlation does not predict patient outcomes [132]. In advanced sarcoidosis, CCL3 correlates with CD8+ lymphocytes [153]. In bleomycin-treated mice, CCL3 is produced by alveolar macrophages and bronchial epithelial cells [154,155,156,157,158]. Antibodies targeting CCL3 [154] and CCR1 [158] exhibit a reduce mononuclear phagocyte accumulation and fibrosis, improving mouse survival. Mice lacking CCL3 or CCR1, or treated with a CCR1 inhibitor, are protected from lung inflammation and fibrosis [159]. TNF-α and IL-6 modulate CCL3 expression in bleomycin-challenged mice, and inhibition of these cytokines decreases CCL3 expression [160]. CCL3 plays a dual role in pulmonary fibrosis by regulating inflammation and fibrogenesis through leukocyte recruitment and fibrocyte migration [157]. In bleomycin-induced fibrosis, mice deficient in both CCL3 and CCR5 exhibit reduced pulmonary influx of TGF-β1-producing cells and less fibrosis [157]. Evasin-1, a tick-derived chemokine-binding protein with a high affinity for CCL3 [50], reduces CCL3 expression and leukocyte recruitment in bleomycin-induced lung fibrosis [50,156].

CCL4: CCL4 (MIP-1β) binds to CCR1, CCR3, and CCR5 receptors, influencing leukocyte influx [10,16]. The role of CCL4 in IPF is less understood. Elevated CCL4 levels have been observed in the BAL samples of IPF patients, but correlations with neutrophils, DLCO, and PaO2 are not significant [131]. In various stages of sarcoidosis, increased CCL4 levels precede advanced fibrotic stages and correlate with CD4+ and CD8+ lymphocytes [153]. In mice, lung expression of Ccl4 mRNA and Ccl4 protein is induced following bleomycin administration [155,157]. However, the predominant role in the development of pulmonary fibrosis in mice is attributed to Ccl3-Ccr5, as the absence of Ccr1 did not impact disease progression and fibrosis [157]. There is ongoing controversy regarding the effect of anti-Ccr1 treatment on leukocyte accumulation and fibrosis induced by bleomycin, as it has been shown to impact mice survival [158].

CCL5: CCL5 (RANTES) engages CCR5 and CCR1, but also CCR2 and CCR3 receptors, influencing both innate and adaptive immunity, including the Th1 response [10,16,161]. In IPF, BAL samples show increased CCL5 mRNA and protein levels by alveolar macrophages compared to healthy volunteers [162]. In fibrosing alveolitis, macrophages (CD68+) and eosinophils are key sources of CCL5 [163], with CCL5 correlating with eosinophil numbers in pulmonary fibrosis caused by sulfur mustard gas inhalation [164], suggesting a potential role of CCL5 in acting on CCR3 and contributing to eosinophilia during fibrosis. In mice, Ccl5 expression is induced in the lungs following bleomycin instillation [155,156,157]. Elevated Ccl5 levels are associated with the influx of Ccr5+ IFNγ-producing γδ T cells, mitigating lethality and lung fibrosis in Ackr2^-/-^ mice, revealing a potential role of CCL5 counterbalancing pulmonary fibrosis triggered by bleomycin [155]. Further studies are required to clarify the precise role of CCL5 in pulmonary fibrosis.

Ccl6: Ccl6 (C10/MRP-1), which binds to the Ccr1 receptor, is associated with the Th2 immune response [10,16]. Elevated levels of Ccl6 are observed during the pathogenesis of bleomycin-induced pulmonary fibrosis [165], and its expression is induced by the Th2 cytokine IL-13 [166]. Neutralizing IL-13 attenuates both bleomycin-induced pulmonary fibrosis and Ccl6 levels [166], suggesting a role for Ccl6 in the development of fibrosis. However, clinical studies on Ccl6 in IPF patients are lacking, as Ccl6 is present in mice but not in humans [16].

CCL7: CCL7 (MCP-3), primarily binding to CCR2, CCR1, and CCR3, acts as a natural antagonist to CCR5 and is associated with a Th2 immune response [10,16]. The role of CCL7 in IPF remains unclear. However, its elevated expression in usual interstitial pneumonia (UIP) biopsies and pulmonary fibroblasts suggests a potential role in fibrosis progression [167]. In patients with systemic sclerosis, serum CCL7 levels correlate with the severity of pulmonary fibrosis [168]. In mouse models of lung fibrosis, Ccl7 is produced during both FITC- and bleomycin-induced fibrosis, and Ccr2 deficiency has been shown to protect against fibrosis [169,170]. Further studies are required to clarify CCL7’s specific role and its interactions with various receptors in the pathology of IPF.

CCL8: CCL8 (MCP-2), binding to CCR1 and CCR2, is associated with a Th2 immune response [10,16]. In IPF patients, BAL fluid concentrations of CCL8 are significantly higher compared to controls [161]. CCL8 is expressed by interstitial α-SMA cells and fibroblasts derived from IPF tissue [171], with elevated CCL8 levels correlating with shorter survival, suggesting its potential use for diagnostic prediction in IPF [171]. As CCL8 binds to CCR1 and CCR2, its effects involve the accumulation of CCR1+ inflammatory cells [158] and CCR2+ macrophages and fibrocytes/fibroblasts, which contribute to collagen deposition [138], indicating a significant role in pulmonary fibrosis.

Ccl9: Ccl9 (MIP-1γ/MRP-2) binds to CCR1 [10,16] and CCL23 is its human orthologue [16]. Ccl9 is found in Gr-1+CD11b+ immature myeloid cells and in the pre-metastatic lung of tumor-bearing mice, with its expression induced by TGF-β signaling [172]. Immature myeloid cells (iMCs) are recruited from the bone marrow to the tumor invasion front express matrix metalloproteinases MMP9, MMP2, and CCR1, migrating toward the Ccl9 expressed by the tumor epithelium in adenocarcinoma [173]. However, no experimental studies on the role of Ccl9 in mouse models of pulmonary fibrosis currently exist.

CCL11: CCL11 (Eotaxin) binds to CCR3 but also to CCR5, which is linked to the Th2 immune response and eosinophil trafficking, acting as a natural antagonist for CCR2 [10,16]. CCR3 is constitutively expressed in lung and bronchial fibroblasts. CCL11 selectively modulates fibroblast activities, including proliferation and collagen synthesis, without affecting myofibroblast differentiation or contractility [174]. It enhances lung fibroblast migration in vitro [174]. BAL CCL11 levels significantly correlate with eosinophil numbers in sulfur mustard gas-induced pulmonary fibrosis, indicating its role in eosinophil recruitment during lung fibrosis [154]. Bleomycin stimulation induces CCL11 production in human lung fibroblasts and epithelial cells in vitro [175], suggesting their involvement in eosinophil recruitment in bleomycin-induced pulmonary fibrosis. In mice, bleomycin instillation induces marked pulmonary expression of Ccl11 and Ccr3 [155,176]. Immunostaining for Ccr3 reveals its expression by eosinophils and neutrophils [176]. Ccl11-deficient mice and neutralizing Ccr3 reduce pulmonary fibrosis, decreasing eosinophilia and neutrophilia, and profibrotic cytokine [176]. IL-13 transgenic mice deficient in Ccr3 show reduced lung eosinophils, attenuating IL-13-mediated mucus cell metaplasia and collagen deposition [177].

Ccl12: Ccl12 (MCP-5), homologous to human CCL2, binds to CCR2 and is associated with innate immunity [10,16]. Induced by bleomycin, Ccl12 may contribute to pulmonary fibrosis. Mice with lung epithelial cell-specific deletion of Ccl12 are protected from bleomycin-induced fibrosis, showing decreased macrophage recruitment with Ccl2 and Ccl7 expression similar to control mice [170]. Pirfenidone reduces fibrocyte numbers in bleomycin-induced fibrosis by decreasing Ccl12 levels [145]. *Gamma-herpesvirus* infection exacerbates fibrosis by up-regulating Ccl12, which is relevant for fibrocyte recruitment via Ccr2 [146]. Targeting Ccl12 may be an antifibrotic strategy, as the Ccl12-Ccr2 axis drives fibrocyte influx and fibroproliferation in mouse fibrosis models, with Ccl12 neutralization being more effective than Ccl2 in protecting from FITC-induced fibrosis [169]. Increased levels of Ccl12 may drive the Ccr2+ IFNγ-producing γδ T cell, reducing pulmonary fibrosis triggered by bleomycin in Ackr2^-/-^ mice [155]. However, clinical studies on Ccl12 in IPF patients are lacking due to its mouse-specific nature [16].

CCL13: CCL13 (MCP-4) binds to CCR1, CCR2, and CCR3, and is linked to the Th2 immune response [10,16]. Limited research on CCL13 in pulmonary fibrosis identifies blood CCL13 levels as a prognostic biomarker in IPF patients treated with placebo versus those treated with pirfenidone [178]. Experimental studies on the role of CCL13 in pulmonary fibrosis are lacking due to its human-specific expression and absence in mice [16].

CCL14: CCL14 (HCC-1) binds to CCR1 and CCR5, and is associated with innate immunity [10,16]. Its role in IPF is unclear, although increased expression has been observed in the ciliated epithelium-enriched subset of IPF patients [179]. However, its impact on the bleomycin mouse model and its effects on fibroblast activities remain unexplored.

CCL15: CCL15 (HCC-2) binds to CCR1 and CCR3, and is associated with innate immunity [10,16]. Its role in IPF is poorly studied. It is primarily produced by ciliated epithelial cells, with higher expression observed in ciliated epithelium-enriched subsets of IPF patients [179]. CCL15 levels in IPF lung tissue and BAL are similar to those in controls but are increased in chronic hypersensitivity pneumonitis (CHP) [180], suggesting its potential as a prognostic biomarker. The CCL15-CCR3 axis is implicated in fibroblast biology, influencing proliferation and TIMP-1 regulation in human gingival fibroblasts [181], but its role in fibrosis remains unexplored.

CCL16: CCL16 (HCC-4) binds to CCR1, CCR2, CCR5, and CCR8, and is linked to monocyte and dendritic cell maturation [10,16]. Its role in IPF remains unexplored, although it has been detected in IPF BAL samples. CCL16 levels are higher in eosinophilic pneumonia compared to sarcoidosis and IPF [182]. However, its specific role in the bleomycin mice model or its effects in vitro on fibroblasts remain unexplored.

CCL17: CCL17 (TARC), recognized by CCR4, is linked to Th2 immune responses [10,16]. It is detectable in IPF BAL fluid and expressed by hyperplastic epithelial cells [183] and CD206+ alveolar macrophages [184], and is correlated with CCR4+ neutrophils, eosinophil influx, and CCR4+ alveolar macrophages [183,185]. Elevated levels of CCL17 in BAL fluid might predict poor outcomes in IPF patients [132]. IPF patients exhibit increased lung CD4+ and CCR4+ CD4+ T cells [186]. CCL17 is a biomarker of pulmonary function decline in chronic hypersensitivity pneumonitis (HP) with fibrosis, serving as a predictor of worsening lung function [187]. Ligand-receptor analyses suggest the CCL17-CCR4 axis mediates chemoattraction in myeloid-enriched IPF subsets [179], and these subsets are responsive to pirfenidone. Isolated BAL leukocytes from IPF patients produce elevated levels of CCL17, particularly in cases of acute exacerbation of IPF (AEIPF) cases [188]. CCL17 drives fibroblast activation in pulmonary fibrosis progression, enhancing TGF-β/Smad signaling [189]. Elevated CCL17 levels in IPF patients and mice with bleomycin-induced pulmonary fibrosis (PF) indicate that the antibody-mediated CCL17 blockade protects against fibrosis [189]. Ccl17 elevation in mice [155,190] is associated with Ccr4+ macrophage accumulation, and Ccr4 deficiency inhibits lung migration and ROS production, attenuating fibrosis [190,191]. Ccl17 enhances wound healing through Ccr4-mediated fibroblast activation and migration in vitro [192]. The CCL17-CCR4 axis is implicated in IPF progression, suggesting potential benefits from targeting CCL17 or CCR4 in fibroproliferative lung diseases.

CCL18: CCL18 (PARC) is a ligand of the PITPNM3/Nir1 receptor (not a member of the GPCR superfamily) but also acts by binding to CCR8, and is related to DC chemoattraction of T and B cells. However, CCL18 does not have a murine homolog [10,16]. Serum CCL18 concentrations have predictive value in IPF and may be a helpful tool for treating IPF patients. There is a higher incidence of disease progression and significantly higher mortality in the group of IPF patients with high serum CCL18 concentrations [193,194]. Blood CCL18 concentrations are a consistent predictor of disease progression across IPF cohorts, and CCL18 was prognostic for absolute change in the percentage of FVC% predicted [178]. Indeed, CCL18 is predictive for the outcomes of ILDs, with higher predictive values for CCL18 in both IPF and systemic sclerosis [195]. The genetic variation of the CCL18 gene also influences CCL18 expression and survival in IPF. Serum CCL18 levels were impacted by the rs2015086 C>T genotype, with the highest CCL18 levels in the presence of the C-allele, and survival was worse with the CT genotype compared to the TT genotype [194,196]. CCL18 levels are increased in serum, BALF, and alveolar macrophage culture supernatant from inflammatory and fibrotic ILDs [197]. Induction of CCL18 production by BAL-derived cells was increased in patients with IPF and correlated negatively with pulmonary function test parameters [198]. CCL18 levels in BAL were negatively correlated with the DLCO, whereas there was a positive correlation between CCL18 levels and neutrophil and eosinophil cell counts in BAL [199]. In a cohort of IPF patients during sleep and exercise, ventilation and gas exchange were compared, where CCL18 negatively correlated with DLCO, arterial oxygen (PaO2), and mean arterial carbon dioxide (PaCO2) during exercise [200]. Furthermore, high levels of CCL18 production by BAL cells significantly predicted the development of AEIPF [188]. Flow cytometry revealed an increase in CCL18+ alveolar macrophages in patients with fibrotic lung diseases [199]. Myofibroblasts derived from fibrocytes highly expressed soluble collagen and CCL18 [136], contributing to tissue fibrosis. Stimulation with collagen type I and III significantly up-regulated CCL18 production by alveolar macrophages derived from IPF, shifting alveolar macrophages to the profibrotic M2, and might perpetuate pulmonary fibrosis [201]. Indeed, culture supernatants of alveolar macrophages from patients with IPF increased collagen production by normal lung fibroblasts and are mediated by CCL18 [198] and PKC-α but not PKC-δ or PKC-ε which mediate the profibrotic effects of CCL18 [202]. Moreover, collagen production by fibroblasts induced by CCL18 requires Sp1 signaling and Smad3 activity [203]. TGF-β-induced CCR8 promoted macrophage transdifferentiation into myofibroblast-like cells mainly through autophagy [125]. CCL18-induced intracellular signaling led to activation of ERK1/2 in fibroblasts, but not p38 [204]. Therefore, the blockage of CCL18 signaling, such as PKC-α, Sp1, Smad3, and ERK1/2 in fibroblasts, may represent targets for antifibrotic therapies. Finally, CCL18 transfection using an adenovirus vector delivery in mice lungs promotes selective and long-term pulmonary infiltration of T lymphocytes and collagen accumulation through a TGF-β-dependent mechanism in mice [205].

CCL19: CCL19 (MIP-3β/ELC) binds to CCR7 and is related to T cells and DC homing to lymph nodes [10,16]. Studies evaluating the role of CCL19 in the context of IPF are scarce. Hyperplastic alveolar epithelial cells, endothelial cells in lymphoid follicles, fibroblasts from fibroblastic foci, and smooth muscle cells expressed CCL19 in IPF lung samples [206]. Ccr7^-/-^ mice failed to mount a fibrotic pulmonary response to bleomycin exposure [207]. Moreover, DCs expressing CCR7+ are observed in lymphoid follicles, suggesting DC recruitment by CCR7 ligands in IPF [206]. The loss of Ccl19 leads to impaired iBALT formation and B- and T-cell responses to influenza infection in mice, suggesting that Ccl19 expression in the lungs plays a crucial role in organizing lymphoid tissues. In lung samples of idiopathic interstitial pneumonia, CCL19 is elevated, and CCR7 protein was expressed in interstitial areas restricted to blood vessels and mononuclear cells [208]. The lymphatic endothelium is the source of CCL19, and CCR7 expression is localized in the lymphatic endothelium. CCR7 was also expressed by CD68+ macrophages around the newly formed lymphatics of lung samples from idiopathic diffuse alveolar damage, suggesting that the CCL19-CCR7 axis may be involved in lymphangiogenesis [209]. Indeed, CCR7 stimulation with CCL19 resulted in significantly increased secretion of VEGF by fibroblast-like synoviocytes and could contribute to angiogenesis [210].

CCL20: The CCL20 (MIP-3α/LARC) is a ligand of CCR6 and is related to T cells and DC homing to lymph nodes [10,16]. The role of CCL20 in the context of IPF is poorly understood. Epithelial cells express CCL20, which is related to CCR6+ lymphocyte infiltration [206], but no DCs expressing CCR6 were observed within the hyperplastic epithelium of the lung sections from IPF patients. In zones of active lung disease, IPF samples showed an increased number of CCR6+ and IL-17A+ expressing cells compared with histologically normal lung areas [211]. However, compared to controls, IPF patients had a lower proportion of CCR6+ CD4+ T cells [186]. CCL20 may contribute to the development of pulmonary arterial hypertension in systemic sclerosis. In patients, serum CCL20 levels correlated inversely with the percentage of predicted DLCO and positively with mean pulmonary artery pressure [212]. CCL20 is produced by airway epithelia and is increased during cystic fibrosis, as revealed by abundant CCL20 levels in BAL fluid from patients with cystic fibrosis [213]. In the blood of cystic fibrosis patients, low circulating CCR6+ ILC2s correlate with increased disease severity and advanced pulmonary disease. However, there is an increased eosinophil, neutrophil, and ILC2 homing into the chronically inflamed lungs of these patients [214]. Mechanistically, ILC2 activation triggers the lung production of type-VI collagen via IL-4 and IL-13, driving extracellular matrix remodeling and respiratory failure [214]. Finally, radiation-induced pulmonary fibrosis leads to Ccr6 expression in the lungs of mice [215] and may be involved in fibrogenesis.

CCL21: CCL21 (6Ckine/SLC) binds to CCR7 and influences T cell and DC homing to lymph nodes [10,16]. In IPF, fibroblast overexpression of CCR7, induced by CCL21, suggests a role in pulmonary fibrosis development [216]. IPF fibroblasts show migratory and proliferative responses to CCL21, inhibited by CCR7-neutralizing antibodies [216]. Lymphatic endothelial cells and lymphocytes localized in lymphoid follicles also express CCL21 [206]. Bleomycin-induced pulmonary fibrosis in mice involves CCL21 induction, with chemotactic responses of CCR7+ bone marrow progenitor cells, and Ccr7^-/-^ mice are protected from fibrosis [207,217]. Adoptive transfer of nonspecific interstitial pneumonia fibroblasts into SCID mice caused diffuse interstitial fibrosis, and systemic immunoneutralization of CCR7 or Ccl21-attenuated pulmonary fibrosis [218].

CCL22: CCL22 (MDC), recognized by CCR4, is associated with Th2 responses [10,16]. It is expressed in hyperplastic epithelial cells and fibroblasts within fibroblastic foci of active fibrosis in IPF samples [206], with CCL22 expression near blood vessels and lymphoid follicles by lymphocytes and high endothelial venules [206]. Elevated CCL22 levels in BAL of IPF patients recruit and activate CCR4+ alveolar macrophages [183,219]. Alveolar macrophages from lung fibrosis patients spontaneously produce CCL22 and exhibit increased CD206 expression, an M2 marker [184]. BAL fluid CCL22 levels correlate with CCR4-expressing alveolar macrophages but not with total cell numbers, alveolar lymphocytes, or macrophages in IPF BAL fluid [183]. Clinically, CCL22 BAL fluid levels inversely correlated with diffusion lung capacity for carbon monoxide/alveolar ventilation per minute (DLCO/VA) values in IPF patients [183] and the percentage of BAL CCR4+CD4+ lymphocytes negatively correlated with DLCO [185]. Therefore, this suggests that the local overexpression of CCL22 may induce lung dysfunction by recruiting and activating CCR4-positive alveolar macrophages and lymphocytes. Elevated levels of CCL22 in BAL fluid might be predictive of a poor outcome in patients with IPF [132] and are also elevated in patients with an AEIPF [188]. In mice, Ccl22 is produced during the pathogenesis of bleomycin-induced pulmonary fibrosis [155,190] and Ccr4^-/-^ mice are protected from pulmonary fibrosis [207].

CCL23: CCL23 (MPIF-1) initially binds to CCR1 with low affinity, potentially increasing via proteolytic modification during inflammation in vivo [10,16]. Its role in IPF is unclear. Serum CCL23 levels are elevated in systemic sclerosis, correlating with higher pulmonary arterial hypertension rates [220]. Human neutrophils produce CCL23 in response to TLR agonists or TNF-α [221]. CCL23 promotes angiogenesis, enhancing chemotactic migration and endothelial cell differentiation [222] through MMP-2 up-regulation [223]. This suggests potential involvement of CCL23 in tissue remodeling during IPF manifestation.

CCL24: CCL24 (Eotaxin-2) binds to CCR3 and is related to Th2 immune response and eosinophil migration [10,16]. The role of CCL24 in the context of IPF is unclear. Results of BALF proteomics from IPF samples show an up-regulation of CCL24 [224], suggesting the role of this chemokine as a mediator of pulmonary fibrosis. CCL24 stimulates human lung fibroblast proliferation and collagen synthesis; however, it does not induce the expression of α-Smooth Muscle Actin (α-SMA) or TGF-β from lung fibroblasts [225]. Circulating levels of CCL24 are elevated in systemic sclerosis patients [226]. In bleomycin-induced experimental animal models, the blockade of CCL24 with CM-101 profoundly inhibited pulmonary fibrosis and inflammation [226]. Moreover, CCR3 antagonism prevents the infiltration of eosinophils into the airways and the development of allergen-induced subepithelial and peri-bronchial fibrosis [227]. Thus, inhibition of CCL24 can potentially be beneficial for therapeutic use in IPF.

CCL25: CCL25 (TECK) is recognized by CCR9 and is related to thymocyte migration [10,16]; however, the role of CCL25 in the context of IPF is unexplored. CCL25 is markedly up-regulated in plasma from IPF patients [228]. In the lungs, airway eosinophils had decreased CCR3 and increased CCR9 expression in allergic subjects compared with their circulating counterparts [229]. Ccr9 and Ccl25 expressions are induced in airway inflammation and are essential in regulating eosinophil and lymphocyte recruitment inflammation in asthma in mice [230]. The expression of CCR9 is also increased in lung adenocarcinoma compared with normal lungs, and the expression was positively correlated with tumor size and lymph node metastasis and predicts the poor prognosis in patients with lung adenocarcinoma [231]. The CCL25 activation of CCR9 promotes breast tumor cell migration, invasion, and MMP expression, which is involved in metastization [232]. Further studies are needed to elucidate CCL25’s role in IPF.

CCL26: CCL26 (Eotaxin-3) binds to CCR3 and is related to the Th2 immune response but interacts with CCR1, CCR2, and CCR5 as a natural antagonist [10,16]. The role of CCL26 in IPF is still obscure. Despite CCL24 and CCL26 being recognized by CCR3, they exert differential profibrogenic effects on human lung fibroblasts. Unlike CCL24, CCL26 did not stimulate human lung fibroblast proliferation and collagen synthesis but promoted fibroblast chemotaxis in Boyden chambers [225] without inducing α-Smooth Muscle Actin or TGF-β expression by lung fibroblasts.

CCL27: CCL27 (CTACK) is a ligand of CCR10 and is related to the homing of T cells to the skin [10,16]. The role of CCL27 in IPF is partially unknown. A recent study aimed to evaluate serum cytokines/chemokines as potential biomarkers to predict outcomes in IPF patients [233]. CCL27 was tested because no previous study had evaluated CCL27 as a potential biomarker of disease progression and survival in IPF. Serum CCL27 was significantly associated with survival in the validation cohort and still significantly correlated with prognosis after adjusting for age and using antifibrotic drugs [233]. Thus, CCL27 could be helpful as a novel prognostic biomarker of IPF. Increased soluble immune mediators were observed in the sera of asbestos-exposed workers compared to controls, including CCL27 levels, and may be involved in occupational asbestosis [234]. During severe tuberculosis in juvenile rhesus monkeys, it down-regulated many genes in the blood but up-regulated selected genes constituting gene networks of Th17 and Th1 responses, related to the overexpression of genes encoding inflammatory cytokines and receptors, including CCL27 [235]. Burn wound exudates isolated from deep burn wounds contain elevated CCL27, and recombinant human-CCL27 stimulation showed increased proliferation and migration of dermal fibroblasts [236]. Gingiva fibroblasts expressed different chemokine receptors, including CCR10, and the stimulation of the CCL27-CCR10 axis up-regulated HGF secretion [181].

CCL28: CCL28 (MEC) recognizes CCR10 and is related to the homing of T cells to mucosal surfaces [10,16]. The role of CCL28 in the context of IPF is undetermined. CCL28, a mucosa-associated chemokine, is present in normal human lungs, monkey lungs, and several asthmatic lung tissues, suggesting that CCL28 is a homeostatic chemokine but may also participate in inflammatory responses associated with the pulmonary mucosal immune system [237]. Indeed, Ccl28 is constitutively expressed in lung tissue collected from non-sensitized control mice, but increased Ccl28 levels were found in mice sensitized and challenged with cockroach antigen, suggesting that Ccl28 seems to regulate eosinophil recruitment to peri-bronchial regions of the lung and eosinophilia [238]. CCL28 is strongly up-regulated in IPF plasma compared to healthy subjects [228]. In lung samples, CCL28 and its receptor CCR10 were both elevated in IPF, and CCR10 was highly expressed by various cells, most notably in mesenchymal progenitor cells (MPCs) [239]. Intravenous injection of human CCR10+ cells initiated and maintained fibrosis in immunodeficient (NOD/SCID-γ) NSG mice. Thus, human CCR10+ cells promote pulmonary fibrosis, and eliminating these cells inhibits lung fibrosis. As fibroblasts express CCR10 in the gingiva, the CCL28-CCR10 axis stimulates fibroblast proliferation and up-regulation of IL-6 and HGF, suggesting that CCL28 has a predominant role in oral wound healing by increasing fibroblast proliferation, migration, and the secretion of IL-6 and HGF, as well as reducing the secretion of TIMP-1 [181]. Thus, the CCL28-CCR10 axis could represent an important target to treat fibrogenesis.

### 4.2. CXC Chemokines in the Context of Pulmonary Fibrosis

CXCL1: CXCL1 (GROα/KC) binds to CXCR2 and is related to neutrophil trafficking and angiogenesis [10,16,240]. The levels of CXCL1 in serum and BALF from IPF patients are increased compared to healthy subjects [241,242]. In patients with AEIPF, the percentage of BAL neutrophils significantly increased compared to stable patients, which is related to increased production of CXCL1 [188]. Elevated CXCL1 levels in BAL are associated with the pro-angiogenic profile during IPF [241]. IPF patients treated with IFN-γ-1b had significantly decreased CXCL1 BAL levels after 12 months of treatment, suggesting that IFN-γ may be an essential mediator counterbalancing angiogenesis during IPF [242]. The CXCL1-CXCR2 axis appears to be involved in the progression of interstitial pneumonia with autoimmune features (IPAF), and CXCL1 indicates disease activity and prognosis. Increased CXCL1 plasma levels were identified in IPAF compared to healthy controls and were clinically associated with low DLCO and involved parenchyma extension [243]. Increased CXCL1 concentrations in BALF were related to increased neutrophil counts in IPAF [243]. Furthermore, circulating CXCL1 levels increased in IPAF patients with acute exacerbations [243]. Cryptogenic fibrosing alveolitis (CFA) is characterized by increased pulmonary recruitment of peripheral blood neutrophils, and plasma levels of CXCL1 were elevated in patients with CFA and may contribute to neutrophilic cryptogenic fibrosing alveolitis [244]. Bleomycin-induced Cxcl1 production in lungs and Cxcl1 levels correlated with neutrophilic airway inflammation in mice preceding pulmonary fibrosis [23,245,246,247] which were attenuated by the Cxcr2 antagonists [245,246] or endothelial glycosaminoglycans antagonism [23]. Particulate matter instillation increased the severity of bleomycin-induced pulmonary fibrosis and, depending on Cxcl1-mediated neutrophil chemotaxis, increased the severity of pulmonary fibrosis [248]. The genetic Cxcl1 deletion or pharmaceutical inhibition of Cxcl1 binding to Cxcr2 by Reparixin ameliorated the exacerbation of pulmonary fibrosis induced by particulate matter [248]. The Cxcr2 antagonist administration reduced the angiogenic activity of human umbilical vein endothelial cells (HUVECs) in vitro and in vivo reduced the lung expression of von Willebrand Factor, a marker of angiogenesis [245] in response to bleomycin. In mice, neutralization of Cxcl1 and Cxcr2 inhibited angiogenic activity, suggesting that the Cxcl1-Cxcr2 axis mediates pulmonary angiogenesis [249]. Cxcl1 can recruit Cxcr2+ endothelial progenitor cells into the lungs of allergen-sensitized mice and lung angiogenic response [250]. In summary, CXCL1 critically mediates neutrophilic inflammation and angiogenesis in pulmonary fibrosis development.

CXCL2: CXCL2 (GROβ/MIP-2α/MIP-2) binds to CXCR2 and is related to neutrophil trafficking and angiogenesis [10,16,240]. The role of CXCL2 in the context of IPF is unknown. Type II alveolar epithelial cells from rat lungs produce the Cxcl2 and express the Cxcr2 [251]. Moreover, rat Cxcl2 stimulated the proliferation of alveolar epithelial cells in vitro [252]. Type I and II epithelial cells from rat lungs secreted Cxcl2 in response to an inflammatory stimulus, such as IL-1β or lipopolysaccharide, in vitro [253]. Lung epithelial cells derived from mice up-regulated Cxcl2 in response to lipopolysaccharide and *Chlamydia pneumoniae* infection in vitro, possibly being involved in neutrophil recruitment associated with ectopic lymphoid tissue formation in mice [254]. Alveolar macrophages are sentinel cells that produce chemoattractant Cxcl2 in the context of pulmonary infection [255], and Cxcl2 is also produced after apoptotic cell phagocytosis [256] potentially sustaining lung inflammation. CXCL2-induced airway smooth muscle cell migration depends on p38 MAPK and CXCR2 [257]. In the mice model of bleomycin-induced fibrosis, Cxcl2 is induced in the lungs [245,246,258]. The presence of Cxcl2 was correlated with a more significant angiogenic response and collagen content in the lungs. Cxcl2 depletion in vivo significantly reduced bleomycin-induced pulmonary fibrosis without changes in pulmonary neutrophil influx, fibroblast proliferation, or collagen gene expression [258]. Warheit-Niemi and colleagues recently described alterations to the trafficking and function of neutrophils after the development of fibrosis induced by bleomycin [259], observing an increased number of aged neutrophils in peripheral tissues of fibrotic mice, driven by an up-regulation of neutrophil chemokine Cxcl2 by lung cells. Cxcl2 can also recruit Cxcr2+ endothelial progenitor cells into the lungs of allergen-sensitized mice [250] and CXCL2-CXCR2 in asthma patients [260]. Indeed, Cxcr2 antagonism attenuated lung angiogenesis and fibrosis in mice challenged with bleomycin [245], supporting that Cxcl2 is an important angiogenic factor that may regulate pulmonary fibrosis development in mice.

CXCL3: CXCL3 (GROγ/MIP-2β) is recognized by CXCR2 and is related to neutrophil trafficking and angiogenesis [10,16,240]. The role of CXCL3 in the context of IPF is not well understood. Expression of CXCL3 is constitutive in human airway epithelial cells and alveolar macrophages, and CXCL3 increases upon stimulation [261]. Airway smooth muscle cells stimulated with IL-17 induce CXCL3 production, and CXCL3 promotes airway smooth muscle cell migration mediated through the CXCR2 receptor [262]. CXCL3-induced migration is mediated by p38 and ERK1/2 MAPK pathways via CXCR2 [257]. Cxcr2 contributes to pulmonary systemic angiogenesis in mice [263], and Cxcl3 may play a role in this process. Cxcl3 may be critical for perpetuating rhinovirus-induced asthma exacerbation through the recruitment of Cxcr2+ neutrophils and by promoting type 2 inflammation [264]. Overall, CXCL3 may contribute to chronic inflammation, angiogenesis, and airway remodeling.

CXCL4: CXCL4 (PF4) recognized by CXCR3 exhibits procoagulant and angiostatic activities [10,16,240]. In IPF, the role of CXCL4 is partially unknown. Elevated Cxcl4 levels are observed in inflammatory and fibrotic mouse models in the skin, lungs, and heart. Cxcl4-deficient mice demonstrate its essential role in promoting lung fibrosis, with overexpression aggravating and blocking, reducing bleomycin-induced fibrosis [265]. Single-cell analysis predicts strong interactions between Cxcl4 produced by macrophages and receptors on endothelial cells, fibroblasts, and alveolar type 2 cells [265]. CXCL4 directly induces human myofibroblast differentiation and collagen synthesis in vitro [265] suggesting its relevance in pulmonary fibrosis and potential as a therapeutic target. CXCL4 and variant CXCL4L1 are angiostatic chemokines activating CXCR3 on microvascular and lymphatic human endothelial cells, countering CXCL12-induced angiogenic signals [266]. CXCL4 is elevated in systemic sclerosis patients and correlates with the presence and progression of disease complications, including lung fibrosis [267], making it a potential disease biomarker. Increased levels of CXCL4 were found in patients with SSc-ILD compared with controls and decreased in patients treated with immunosuppressive therapy. Patients with an increased decline in CXCL4 levels during the first 12 months of immunosuppressive therapy had an improved course of forced vital capacity %-predicted from 12 to 24 months, suggesting that it may have a significant predictive effect on disease progression [268]. CXCL4 is secreted by plasmacytoid dendritic cells in systemic sclerosis [267]. CXCL4 can alter monocyte differentiation, inducing pro-inflammatory and profibrotic phenotypes that directly trigger the fibrotic cascade by producing extracellular matrix molecules and inducing myofibroblast differentiation [269]. Indeed, CXCL4 can drive fibroblast activation indirectly via PDGF-BB production by myeloid cells from systemic sclerosis patients [270]. Recently, it has been reported that CXCL4 also binds to CCR1, inducing CCR1 endocytosis and chemotaxis of primary human monocytes dependent on CCR1 [271]. Bleomycin-induced CXCL4 lung production in mice lungs after instillation, and treatment with an anti-platelet agent decreased CXCL4 release and the formation of platelet-neutrophil aggregates in the lungs, subsequently reducing fibrosis [272]. This suggests the essential role of CXCL4 in promoting fibrotic events.

CXCL5: CXCL5 (ENA-78/LIX) recognizes CXCR1 and CXCR2 and is associated with neutrophil trafficking and angiogenesis [10,16,240]. Hyperplasic type II pneumocytes and macrophages in lung tissues from IPF patients produce CXCL5 in higher amounts compared to control subjects, displaying pro-angiogenic activity [273]. CXCL5 levels in BALF were significantly elevated in IPF patients compared to those with idiopathic nonspecific interstitial pneumonia (NSIP) and healthy controls [274]. Quantifying CXCL5 may help distinguish IPF from other NSIP profiles. Furthermore, CXCL5 BALF levels in IPF patients were higher compared to those in sarcoidosis patients [241]. In IPF, patients treated with IFN-γ for 12 months exhibited reduced CXCL5 levels in BAL [242,275], suggesting that CXCL5 activity may be a critical angiogenic factor in IPF [242,273,275]. CXCL5 levels are correlated with the number of neutrophils in BAL fluid, and increased CXCL5 levels in airspace may be associated with emphysematous lung manifestations in patients with pulmonary fibrosis [276]. The CXCL5-CXCR2 axis was associated with eosinophilia during asthma exacerbations [277]. In mice, platelets and lung-resident cells serve as sources of homeostatic Cxcl5 in blood. Inflammatory Cxcl5 binds erythrocyte Ackr1, impairing Cxcl1 and Cxcl2 scavenging in blood [278], affecting neutrophil migration in the lungs.

CXCL6: CXCL6 (CGP-2) is a ligand of CXCR1 and CXCR2, associated with neutrophil trafficking and angiogenesis [10,16,240]. The role of CXCL6 in the context of IPF is not fully elucidated. Human bronchial epithelial cells release CXCL6 in response to IL-17 [279]. Elevated levels of CXCL6 were detected in BALF samples from patients with IPF [247]. In patients with systemic sclerosis, serum CXCL6 levels positively correlated with the severity of pulmonary fibrosis and were elevated in association with pulmonary vascular involvement [280]. Cxcl6 is induced by bleomycin instillation in mice, and anti-Cxcl6 blockade attenuated acute pulmonary neutrophil influx and cytokine production. In the later phase, it also attenuated lymphocyte recruitment, collagen deposition, and fibrotic lesions in the lungs [247]. Despite its role in neutrophil migration, Cxcl6 is also associated with angiogenesis and tumor proregression in mice [281], but its specific role in lung angiogenesis tissue is not fully described.

CXCL7: CXCL7 (NAP-2) is recognized by CXCR2 and is associated with neutrophil trafficking and angiogenesis [10,16,240]. The role of CXCL7 in the context of IPF is unknown. Proteomic analysis of BALF for molecular profiling of fibrotic lung diseases revealed elevated levels of CXCL7 in BALF from IPF patients [224], a finding also confirmed by immunohistochemistry of IPF samples [282]. Stimulated murine immortalized alveolar macrophages also produce Cxcl7 [282]. Cxcl7 may contribute to the pathogenesis of acute lung injury (ALI) through neutrophil chemotaxis, vascular activation, and permeability. Cxcl7^-/-^ mice are protected from ALI, preserving endothelial/epithelial barrier function combined with impaired neutrophil transmigration [283]. CXCL7+ immune-stained cells were increased in the bronchial submucosa of patients with stable severe chronic obstructive pulmonary disease (COPD) compared with control non-smokers. These patients also exhibited increased expression of extracellular matrix-binding receptors on neutrophils [284], implying a role in COPD pathogenesis.

CXCL8: CXCL8 (IL-8) acts as a ligand for the CXCR1 and CXCR2 receptors, playing a crucial role in neutrophil trafficking and angiogenesis [10,16,240]. Its significance is well established in the context of IPF, where chronic airway neutrophilia is linked to pathology. Alveolar macrophages, as a source of CXCL8, contribute to the influx of neutrophils into the lungs of IPF patients [130,285,286]. In these patients, elevated CXCL8 levels correlate with bronchoalveolar lavage (BAL) neutrophils [287]. The expression of CXCL8 mRNA by alveolar macrophages and CXCL8 protein levels in BAL samples align with the pattern of airway neutrophilia and disease severity [130,285,286]. Clinical evidence suggests that alveolar macrophages from IPF patients may be primed for CXCL8 overproduction, potentially influencing neutrophilic alveolitis in the subacute phase of IPF [288]. Notably, various cell types, including type II pulmonary epithelial cells, alveolar and interstitial macrophages from patients with idiopathic interstitial pneumonia (IIP), and interstitial pneumonia with collagen vascular disease (IP-CVD), express CXCL8 [289]. Serum levels of CXCL8 in IPF correlate with the degree of neutrophilic alveolitis, indicating its potential as a marker for disease severity [290]. Furthermore, CXCL8 is up-regulated in advanced ILDs with prominent fibrosis and declining lung function [291]. Its serum levels negatively correlate with indicators such as DLCO, total lung capacity (TLC), FVC, and PaO2 [290]. Additionally, CXCL8 serum levels in IPF samples correlate negatively with fast expiratory volume in 1s (FEV1%) and FVC% [292]. An exaggerated expression of CXCL8 in BAL-recovered immune cells is a common characteristic in both IPF and sarcoidosis patients with progressing disease [152]. Single nucleotide polymorphisms (SNPs) in the CXCL8 gene increase the risk of IPF, with a promoter SNP (rs4073T>A) common allele potentially contributing to IPF development through CXCL8 up-regulation [293]. High plasma concentrations of CXCL8 are associated with poor overall survival, poor transplant-free survival, and poor progression-free survival in IPF patients [294]. During AEIPF, CXCL8 production increases, contributing to elevated neutrophil numbers in BAL compared to stable IPF patients [188]. High CXCL8 levels characterize early IPF acute exacerbation, with increased levels predicting worse outcomes [295]. Furthermore, increased CXCL8 levels in airspaces may be linked to emphysematous lung changes in pulmonary fibrosis patients. CXCL8 levels inversely correlate with vital capacity, DLCO/VA, and positively correlate with composite physiological index (CPI) and extent of areas of low attenuation on CT [276]. Endothelial progenitor cells from IPF patients produce CXCL8, contributing to neutrophil invasion during IPF [296]. The CXCL8-CXCR1/2 axis in endothelial cells is implicated in lung fibrosis pathogenesis and angiogenesis. Immunolocalization shows that pulmonary fibroblasts from IPF patients are the predominant cellular source of CXCL8, with CXCL8 exhibiting angiogenic activity in IPF progression [287,297]. CXCL8 concentrations in BALF correlate with fibrosis extension, potentially serving as an early-phase marker for IPF [298]. Treatment with IFN-γ-1b reduces BAL CXCL8 levels in IPF patients, indicating a potential role for IFN-γ as a modulator of angiogenic pathways during IPF [242]. CXCL8 may act as a mediator of IPF fibrogenesis, driving fibrotic progression. TGF-β1 induces CXCL8 expression and production in normal human lung fibroblasts (NHLFs) in vitro [299]. In an autocrine manner, CXCL8, through activation of CXCR1, promotes self-renewal and proliferation of fibrogenic mesenchymal progenitor cells (MPCs), potentially serving as a cell of origin for IPF fibroblasts [300]. In a paracrine manner, secreted CXCL8 stimulates macrophage migration in IPF samples, with MPC localization correlating with activated macrophages forming the active fibroblastic focus [300]. Recent data suggest that IL-8 promotes DNA damage-induced senescence and high PD-L1 expression in IPF mesenchymal progenitor cells (MPCs), allowing them to evade immune cell-targeted removal. Disrupting the PD-1-PD-L1 interaction may limit IPF MPC-mediated fibrotic progression [301]. In mice, antagonism of Cxcr1 and Cxcr2 reduces pulmonary fibrosis, acting on endothelial cells, neutrophils, and fibroblasts, and mediating neutrophilic lung inflammation, angiogenesis, and fibrogenesis [245,246,302].

CXCL9: CXCL9 (MIG) is a ligand of CXCR3 and is associated with the Th1 immune response, acting as an angiostatic factor for endothelial cells and functioning as a natural antagonist for CCR3 [10,16]. In IPF, CXCL9 serum levels are up-regulated compared to sarcoidosis and healthy control subjects. However, CXCL9 BALF levels are significantly higher in sarcoidosis patients than in IPF patients [241]. Lower serum CXCL9 serves as an essential predictor of lung function deterioration in IPF patients [187]. Pulmonary hypertension (PH) is a common and severe comorbidity in interstitial lung diseases like IPF, and increased collagen deposition is linked to local NKT cell deficiency [303]. Secretome analysis of peripheral blood mononuclear cells identified CXCL9 and CXCL10 as indicators of NKT cell activation [303]. Pharmacological administration of CXCL9, but not CXCL10, strongly inhibits collagen deposition in isolated human pulmonary arterial smooth muscle cells (hPASMCs) and ex vivo precision-cut lung slices from end-stage IPF-PH patients [303]. This discovery reveals a novel therapeutic strategy for targeting vascular fibrosis in interstitial lung disease. In IPF lung samples, CXCR3 is expressed by type II pneumocytes and fibroblasts in fibrotic areas, as well as in cells undergoing epithelial-to-mesenchymal cell transition [304]. In vitro, CXCL9 prevents TGF-β1-induced epithelial-mesenchymal cell transition in human alveolar epithelial cells [304]. However, IPF patients treated with IFNγ-1b experienced decreased CXCL9 levels [242]. IPF patients exhibit significantly lower CXCR3 expression on BAL CD4 T cells than healthy groups. IPF patients treated with corticosteroids show higher CXCR3 expression correlated with BAL lymphocytes compared to untreated patients [185]. Lower serum CXCL9 in patients with hypersensitivity pneumonitis (HP) is associated with a more significant decline in vital capacity, suggesting that CXCL9 is a crucial predictor of lung function deterioration similar to IPF [187]. In mice, the Cxcl9-Cxcr3 axis acts as a central antifibrotic factor, promoting the expression of the antifibrotic decoy receptor sIL-13Rα2 by pulmonary fibroblasts [305], limiting fibroblast activation and reducing extracellular matrix production.

CXCL10: CXCL10 (IP-10), a CXCR3 ligand linked to the Th1 immune response, acts as an angiostatic factor and natural CCR3 antagonist [10,16]. Control subjects exhibit higher CXCL10 levels than IPF patients in tissue specimens [297], while sarcoidosis patients have higher BALF CXCL10 levels than IPF patients [241]. IFNγ-1b treatment in IPF patients does not alter CXCL10 levels [242], indicating generally low CXCL10 levels in IPF. In a bleomycin-induced pulmonary fibrosis mouse model, Cxcl10 is highly expressed [306,307]. Cxcl10-deficient mice show increased pulmonary fibrosis with enhanced fibroblast migration and lung accumulation [306]. Conversely, transgenic mice overexpressing Cxcl10 are protected from mortality and fibrosis after bleomycin exposure, demonstrating reduced lung fibroblast accumulation [306]. Systemic Cxcl10 administration significantly reduces angiogenesis without altering leukocyte populations [307], indicating its role in inhibiting fibroplasia and extracellular matrix deposition by regulating angiogenesis. Cxcr3-deficient mice exhibit increased mortality with progressive interstitial fibrosis induced by bleomycin, suggesting that Cxcl10-Cxcr3 axis activation limits tissue fibroproliferation by reducing IFN-γ and Cxcl10 production [308]. The Cxcl10-Cxcr3 axis promotes sIL-13Rα2 decoy receptor expression by pulmonary fibroblasts [305], modulating fibroblast-derived extracellular matrix production. Interaction between syndecan-4 and Cxcl10 in the lung interstitial compartment inhibits fibroblast recruitment and pulmonary fibrosis in mice. Recombinant Cxcl10 protein administration inhibits bleomycin-induced pulmonary fibrosis in mice [307,309], but this effect is abrogated in Sdc4^-/-^ mice [309]. Induced pluripotent stem cells (iPSCs) attenuate pulmonary fibrosis, neutrophil accumulation, and improve survival after bleomycin treatment by increasing antifibrotic Cxcl10 production in injured lungs [310].

CXCL11: CXCL11 (ITAC), binding to CXCR3 and associated with the Th1 immune response, acts as an angiostatic factor for endothelial cells and a natural antagonist for CCR3 and CCR5 [10,16]. In IPF, the role of CXCL11 is unclear. IPF patients exhibit basal CXCL11 levels; however, IFN-γ1b treatment increases BAL and serum CXCL11 levels while decreasing elastin, Type III pro-collagen, and PDGF-B in IPF samples [275]. Induced by bleomycin in mice, the Cxcl11-Cxcr3 axis reduces pulmonary collagen deposition, pro-collagen gene expression, histopathologic fibroplasia, and extracellular matrix deposition in the lungs [311]. Cxcl11 treatment also reduces the total number of endothelial cells in the lung following bleomycin exposure [311], inhibiting pulmonary fibrosis by modulating aberrant vascular remodeling.

CXCL12: CXCL12 (SDF-1), a CXCR4 ligand linked to lymphopoiesis and hematopoietic progenitor cell circulation/homing [10,16], may drive tissue fibrosis in IPF by binding to CXCR4. IPF patients show lower CXCR4+ cell numbers but higher CXCL12 levels in the blood than healthy individuals. Elevated CXCR4 expression is detected in IPF and other fibrotic ILDs, primarily in CXCR4-expressing epithelial or myeloid cells [312]. Strong CXCR4 expression is found in vessels near fibrotic areas in biopsy specimens from IPF patients [206,313], with high expression in both epithelial cells and macrophages undergoing fibrotic remodeling [314]. Targeted PET scanning shows CXCR4 up-regulation in fibrotic lung regions, particularly in subpleural honeycombing zones [314]. Early changes in CXCR4 signaling after initiating pirfenidone treatment correlate with the long-term course of FVC after 12 months. Patients with high pulmonary CXCR4 signals on follow-up PET scans after 6 weeks into treatment have a statistically worse outcome at 12 months [314], predicting IPF patient outcomes with pirfenidone. In IPF lungs, CXCL12 is prominently expressed by alveolar epithelial cells [315], and hyperplastic epithelial cells and fibroblasts within fibroblastic foci [206]. A negative correlation is identified between plasma CXCL12 levels, DLCO, and oxygen saturation during exercise in IPF [315]. Circulating fibrocytes, associated with increased blood CXCL12 levels, contribute to tissue fibrosis in IPF patients by intensively remodeling the pulmonary vasculature [315]. Fibrocytes CXCR4+ are elevated in IPF patients associated with high levels of CXCL12, indicating the recruitment of fibroblast and myofibroblast precursors from the circulation to the focus of fibrosis [315]. Fibrocytes isolated from IPF patients’ blood produce CXCL12 and induce endothelial colony-forming cell (ECFC) proliferation and differentiation via the CXCL12-CXCR4 pathway [313]. Human lung fibroblasts (HLFs) produce CXCL12 and show high CXCR4 expression, with CXCL12 stimulating HLF migration and proliferation, enhancing CXCR4 expression [316]. The CXCL12-CXCR4 axis activates the MEKK1/JNK-signaling pathway, leading to SMAD3 phosphorylation and inducing CTGF [317]. In mice, bleomycin induces up-regulation of Cxcr4 and Cxcl12 expression in lung and BAL fluid. Treatment with AMD3100, a Cxcr4 antagonist, significantly reduces lung collagen content, leukocyte infiltration, and fibrocyte infiltration [318,319,320]. Cxcr4 and Cxcl2 are up-regulated in lung tissues of mice exposed to crystalline silica, and blocking Cxcr4 with AMD3100 reduces silica-induced lung injury, collagen deposition, neutrophil infiltration, NETs formation, B-lymphocyte aggregation, and circulating fibrocyte Cxcr4+ recruitment into the lungs [321]. Neutralization of Cxcl12 or blocking the Cxcr4 receptor reduces bleomycin-induced lung fibrosis by diminishing the pulmonary influx of circulating fibrocytes [217,322,323,324]. Loss of Twist1 in mesenchymal collagen-producing cells increases bleomycin-induced pulmonary fibrosis by up-regulating Cxcl12 expression in mice [325], highlighting the crucial role of the CXCR4-CXCL12 axis in IPF pathogenesis.

CXCL13: The CXCL13 (BLC) binds to CXCR5, a homeostatic receptor involved in B and T-cell trafficking in lymphoid tissues [10,16]. CXCL13 is produced by pulmonary tissues during IPF, but which cells are involved remains unclear. CXCL13 is produced by CD68+ and CD206+ alveolar macrophages from patients with IPF, and TNF-α and IL-10 control optimal CXCL13 gene expression in alveolar macrophages, activating the NF-κB and JAK/STAT pathways [326]. In IPF, moderate or high levels of CXCL13 in the blood correlate with annual changes in FVC, DLCO, TLC, and the total ILD lesions, suggesting that CXCL13 may be a predictive biomarker for the outcomes of patients with IPF [318]. CXCL13 is increased in patients with IPF. It is associated with IPF progression and serves as a prognostic biomarker, mediating B-cell trafficking, with its increased dysregulated B cells implicated in IPF pathogenesis [327]. CXCL13 encoded in the lymphoid cluster corresponds to IPF disease severity and shortens survival time according to the Cox proportional hazards model [328]. Moreover, the proportions of T follicular helper (Tfh) cells CXCR5+ PD-1+ ICOS+ increase in the blood of IPF patients compared with healthy controls [329], suggesting that humoral immunity aberration may be implicated in the pathophysiology of IPF. In patients with IPF treated with pirfenidone, CXCL13 was prognostic for progression outcomes in the placebo groups of the test cohort [178]. The role of CXCL13 in the context of mouse models of pulmonary fibrosis is unexplored.

CXCL14: The CXCL14 (BRAK) is associated with macrophage migration, but its receptor is still unknown [10,16]. The role of CXCL14 in the context of IPF is unclear. CXCL14 was observed in Type II alveolar epithelial cells, fibroblast foci, and macrophages in lung samples from IPF. CXCL14 BAL levels correlated with increased macrophages in IPF patients, and serum levels correlated with spirometry parameters, suggesting that CXCL14 may participate in the progression of IPF [330]. CXCL14 was found in IPF lungs but not observed in normal lungs, and it was expressed in regions on the periphery of fibrotic foci and dense fibrosis. CXCL14 may serve as a signal for the α-SMA+ cell recruitment and activation [331]. Transcriptomic analysis through microarray and RNA sequencing (RNA-Seq) revealed that CXCL14 had substantial differential gene expression in IPF lung [123,331,332]. Circulating CXCL14 protein levels are higher in plasma from IPF patients than controls [333], and CXCL14 may represent a systemic biomarker to identify IPF patients. In patients with IPF treated with pirfenidone, CXCL14 was prognostic for progression outcomes in the placebo groups of the test cohort [178].

Cxcl15: Cxcl15 (Lungkine), a murine chemokine linked to neutrophil trafficking with an unknown receptor [10,16], was initially detected in lungs and fetal tissue, suggesting a role in lung development. The secreted Cxcl15 protein induces in vitro and in vivo neutrophil migration, indicating involvement in lung-specific trafficking [334]. Crucial for neutrophil migration from lung parenchyma to airspace, Cxcl15^-/-^ mice are more susceptible to *Klebsiella pneumonia* infection, displaying decreased survival and increased bacterial lung burden [335]. No studies on Cxcl15’s role in bleomycin-induced pulmonary fibrosis or clinical studies with IPF patients exist, as Cxcl15 is only present in mice and not in humans [16].

CXCL16: CXCL16 (SR-PSOX), the CXCR6 ligand linked to NKT and γδ T-cell trafficking and survival [10,16], has an unexplored role in IPF. Constitutively expressed in the bronchial epithelium, CXCL16 may be involved in T-cell recruitment into the lungs in health and disease [336]. Immunohistochemistry suggests alveolar macrophages as the source of CXCL16 in ILDs [337]. Increased CXCR6 expression in lung, relative to blood in ILD patients, associates with elevated CXCL16 in BAL [337]. Serum CXCL16 levels are higher in RA-ILD patients, correlating with lung fibrosis severity [338]. The CXCL16-CXCR6 axis promotes fibrosis through human pulmonary fibroblast proliferation, migration, and collagen production via the PI3K/AKT/FOXO3a-signaling pathway [338]. Cxcl16 induces STAT3 phosphorylation and mouse lung fibroblast proliferation, emphasizing STAT3’s importance in the Cxcl16-Cxcr6 pathway [339]. Up-regulation of CXCL16 and CXCR6 in bleomycin-induced EMT in human alveolar type II epithelial cells promotes pulmonary fibrosis via the TGF-β1/Smad3-signaling pathway [340]. CXCL16 is implicated in lymphocyte accumulation in the lungs, primarily at the respiratory epithelium. Mice lacking CXCL16 exhibit decreased CXCR6+ CD8+ TRM cells in airways, suggesting the CXCR6-CXCL16 axis controls T CD8+ RM cell localization in different lung compartments [341]. The CXCR6-CXCL16 axis is sufficient to drive MAIT cell accumulation in the lungs without infection [342].

CXCL17: CXCL17 (DMC/VCC-1) maintains mucosal homeostasis, regulating myeloid-cell recruitment and angiogenesis [10,16]. Constitutively expressed in mucosal tissues, including lungs, trachea, and bronchus [343,344], CXCL17’s receptor is the GPR35/CXCR8 [345]. In pulmonary fibrosis, up-regulated CXCL17 levels in BALF during IPF suggest its pathogenic involvement [344]. Airway epithelium produces CXCL17 as a chemoattractant for monocytes [345]. Cxcl17^-/-^ mice exhibit significantly fewer lung macrophages in mucosal tissues [346]. CXCL17 demonstrates potent antimicrobial activities, disrupting bacterial membranes through peptides [344]. CXCL17 serum levels rise in pandemic influenza A (H1N1) patients but not in COVID-19 or pulmonary tuberculosis patients [347].

### 4.3. CX3C Chemokine in the Context of Pulmonary Fibrosis

CX3CL1: CX3CL1 (Fractalkine) binds to CX3CR1 and influences monocyte/macrophage, NK, and Th1 cell migration [10,16]. In IPF patients, elevated CX3CL1 correlates positively with CD8+ T cells and negatively with CD4+ T cells in BALF, indicating severity of lung parenchyma impairment [233]. The CX3CL1-CX3CR1 axis is expressed in altered alveolar epithelium, fibrotic tissue, stromal cells, and a subpopulation of epithelial cells [348]. High CX3CR1 in BALF predicts poor prognosis in IPF, influencing NK cell infiltration and survival [349]. CX3CL1 and CX3CR1 are expressed in fibroblast foci, with isolated IPF lung fibroblasts in vitro [348]. CX3CR1 in transitional macrophages correlates with a profibrogenic phenotype in IPF patients [350]. In systemic sclerosis, CX3CR1 and CX3CL1 cooperate with lung manifestations. Elevated CX3CR1 in lung tissues of diffuse systemic sclerosis patients is observed [351]. CX3CR1 may predict SLE with PF, offering a potential treatment target [352]. CX3CL1 on endothelial cells, along with increased soluble CX3CL1, associates with pulmonary fibrosis severity [351]. In systemic sclerosis-related progressive ILD, CX3CL1 levels highly correlate with DLCO [353]. CX3CL1 is linked to type II pneumocytes and airway epithelial cells, while CX3CR1 is expressed by infiltrating interstitial mononuclear cells, especially plasma cells [353]. The CX3CL1-CX3CR1 axis is associated with pulmonary fibrosis in systemic lupus erythematosus (SLE), with increased CX3CR1 in PBMCs predicting disease progression [352]. Lung fibroblasts produce CX3CL1 in response to IL-1β, potentially impacting pulmonary inflammation and fibrosis [354]. Notably, CX3CL1 decreases pro-collagen production from IPF-derived fibroblasts, suggesting an antifibrotic role [348]. In mice, local Cx3cl1 in response to bleomycin promotes pulmonary fibrosis by attracting Cx3cr1-expressing M2 macrophages and fibrocytes. Cx3cr1^-/-^ mice exhibit reduced fibrosis induced by bleomycin, with a skewed macrophage phenotype toward M1 [355]. Cx3cr1+ SiglecF+ transitional macrophages, exerting a profibrotic effect, accumulate in the fibrotic niche and drive fibroblast accumulation and fibrosis after lung injury [350]. In a hyperoxia-induced pulmonary fibrosis mice model, elevated CX3CR1 regulates macrophage autophagy and pulmonary fibrosis through the Akt1-signaling pathway [259]. Ablation of Cx3cr1+ cells decreases fibro-obliteration and myofibroblast accumulation in a model of bronchiolitis obliterans syndrome (BOS) fibrotic disease in mice [356]. CX3CR1 regulates macrophage autophagy, promoting hyperoxia-induced lung injury and pulmonary fibrosis, with silencing alleviating lung fibrosis [357].

### 4.4. XC Chemokines in the Context of Pulmonary Fibrosis

XCL1: XCL1 (Lymphotactin α) binds to XCR1, facilitating CD8+ DC antigen cross-presentation [10,16]. Despite low lung expression [358], XCL1’s role in IPF is unexplored. In pulmonary pathologies, XCL1 is crucial for T lymphocyte migration during chronic tissue remodeling. iNKT cells express high levels of Xcl1 and XCL1, mediating the XCL1-XCR1 axis. This axis in allergic asthma recruits CD103+ DCs into the lungs [359]. In COPD models, elevated Xcl1 correlates with increased CD8+ T cells, altered CD4+/CD8+ cell ratio, and reduced CD4+ T cell activity, indicating involvement in chronic inflammation [360]. In vivo Xcl1 silencing during *Mycobacterium tuberculosis* infection reduces T lymphocytes, IFN-γ response, and increases fibrosis [361], suggesting a contribution to local pathology.

XCL2: The XCL2 (Lymphotactin β) binds to XCR1 and is related to antigen cross-presentation by CD8+ DCs [10,16]. No significant functional difference has been shown between XCL1 and XCL2 [16] and the molecular and functional properties of XCL2 need to be explored. Furthermore, the role of XCL2 in the context of IPF is entirely unknown.

### 4.5. Atypical Chemokine Receptors (ACKR) in the Context of Pulmonary Fibrosis

ACKR1: ACKR1 (DARC/Duffy) is a versatile chemokine receptor, binding to over 20 inflammatory chemokines, including CCL2, CCL5, CCL7, CCL11, CCL13, CCL14, CCL17, CXCL1, CXCL2, CXCL3, CXCL4, CXCL5, CXCL6, CXCL7, CXCL8, CXCL11, and CXCL12. It overlaps with various chemokine receptors such as CCR1, CCR2, CCR3, CCR4, CCR5, CXCR1, CXCR2, CXCR3, and CXCR4. Expressed in erythrocytes, leukocytes, and endothelial cells, ACKR1 is involved in chemokine transcytosis and Plasmodium vivax cell entry into red blood cells [10,16,20,34]. In lung diseases, ACKR1-positive vessels associate with inflammation, increasing during acute lung rejection episodes. ACKR1 expression correlates with an influx of interstitial CCR5-positive T cells and CXCR1-positive leukocytes in acute lung rejection patients [362]. The rs2814778 polymorphism in the ACKR1 gene is linked to worse outcomes in African Americans with acute lung injury, increasing circulating CXCL8 [363]. Cxcl5 ligand modulates Ackr1 scavenger activity, affecting CXCL1 and CXCL2 concentrations, disrupting chemokine gradients, and desensitizing CXCR2. In an Escherichia coli pneumonia model, this leads to increased bacterial burden and mortality [278]. Transfusion of Ackr1 knockout red blood cells into Ackr1 wild-type endotoxemic mice promotes airspace neutrophils, inflammatory cytokines, and lung microvascular permeability compared to transfusion of Ackr1 wild-type red blood cells, highlighting Ackr1’s role in transfusion-related lung inflammation [364]. Recent studies suggest that ACKR1 promotes CXCL12 dimerization [34] impacting their functions, but its specific role in IPF remains unclear.

ACKR2: ACKR2 (D6/CCBP2) is a versatile chemokine scavenger primarily recognizing inflammatory chemokines from the CC and a few from the CXC subfamily (e.g., CCL2, CCL3, CCL4, CCL5, CCL7, CCL8, CCL11, CCL12, CCL13, CCL17, CCL22, CCL23, CCL24, CCL25, CXCL2, and CXCL10). It shows ligand overlap on CCR1, CCR2, CCR3, CCR4, CCR5, CXCR1, CXCR2, and CXCR3 receptors [10,16,20,34]. Constitutively expressed in lymphatic endothelial cells, B cells, placental trophoblasts, and lungs [10,16,34], ACKR2 acts as a negative regulator of inflammatory chemokine responses, notably in lung tissue. Predominantly found in alveolar macrophages (AMs), the percentage of ACKR2+ AMs significantly increases in COPD patients, correlating with reduced lung function parameters (FEV1 and FEV1/FVC) [365]. In advanced COPD, increased ACKR2+ lymphatics are observed in the alveolar parenchyma and bronchioles compared to controls [366]. In septic patients, lungs exhibit increased ACKR2 cells in BAL compared to non-septic patients [367]. Ackr2^-/-^ mice show exacerbated acute lung injury during CLP, marked by increased neutrophil infiltration and elevated CC chemokine levels, leading to reduced survival [367]. However, Ackr2^-/-^ mice are protected from influenza A virus infection (IAV), dampening Ccl5 levels and reducing Th1, Tregs, and B lymphocyte recruitment during IAV infection, impairing pathogen control, and promoting lung dysfunction in wild-type mice [368]. ACKR2’s role in IPF samples is undetermined. Bleomycin-induced pulmonary fibrosis increases Ackr2 expression [155], while CCR4^-/-^ mice, protected from fibrosis, exhibit higher Ackr2 levels [191]. In contrast, Ackr2^-/-^ mice show reduced lethality, lung fibrosis, tissue-remodeling gene expression, leukocyte influx, pulmonary injury, and dysfunction induced by bleomycin [155]. Ackr2^-/-^ mice early exhibit elevated Ccl5, Ccl12, Ccl17, and IFNγ, with increased Ccr2+ and Ccr5+ IFNγ-producing γδ T cells in the airways, counterbalanced by low Th17-lymphocyte influx. Reduced accumulation of IFNγ-producing γδ T cells is observed in Ccr2^-/-^ and Ccr5^-/-^ mice [155]. Ackr2 is expressed by blood endothelial cells [369] and experiments with bone marrow chimeras highlight the pivotal role of endothelial cell Ackr2 expression in bleomycin-induced pulmonary fibrosis [155]. Overall, Ackr2 is a crucial regulator of chemokine-dependent lung tissue inflammatory responses and fibrosis.

ACKR3: ACKR3 (CXCR7/RDC1) is a chemokine scavenger that recognizes homeostatic chemokines (i.e., CXCL11 and CXCL12) with ligand overlap properties on CXCR3 and CXCR4 receptors [10,16,34]. It also binds to the human herpesvirus 8 (HHV-8)-encoded CC chemokine vCCL2/vMIP-II [370]. Furthermore, it has recently been described that ACKR3 is a scavenger receptor for opioid peptides, enkephalins, and dynorphins, reducing their availability for classical opioid receptors [371]. ACKR3 is expressed by vascular endothelial cells, B cells, neurons, and adrenal glands [34]. However, the role of ACKR3 in the context of IPF remains poorly explored. Recently, the potential antifibrotic role of ACKR3 activation was tested. In vivo, administration of the ACKR3 agonist had no inhibitory effect on bleomycin-induced lung fibrosis but had a significant antifibrotic effect in the carbon tetrachloride (CCl4)-induced liver fibrosis model [372]. ACKR3 is highly expressed in most tumor-associated blood vessels and malignant cells from lung cancers but not in normal vasculature [373]. CXCR7 is expressed by the pulmonary endothelium explanted from human hypertensive lungs [374], and in vitro experiments have suggested that CXCR7 might inhibit fibrosis via Wnt/β-catenin pathways during the process of angiogenesis [375]. Therefore, ACKR3 may contribute to endothelial cell proliferation, regeneration, and repair, indicating that it could be critical in pulmonary vascular diseases and fibrosis.

ACKR4: The ACKR4 (CCX-CKR/CCRL1) functions as a chemokine scavenger, recognizing both homeostatic and inflammatory chemokines such as CCL19, CCL21, and CCL25. Recent findings indicate its binding capacity to CCL20 and CCL22 as well [376,377]. These interactions exhibit ligand overlap properties on CCR4, CCR7, CCR6, and CCR9 receptors [16,34]. The expression of ACKR4 is observed in lymphatic endothelium and keratinocytes [16,34]. While the lungs express low levels of ACKR4 [378], it is prominently present in the thymus, specifically in thymic epithelial cells within cortical and subcapsular zones [379]. In this context, ACKR4 suppresses the entry of thymocyte progenitors into the thymus. However, the role of ACKR4 in the context of IPF remains unclear. Notably, ACKR4 transcripts were up-regulated in bronchoalveolar lavage (BAL) cells from sarcoidosis patients, showing localization and association with ciliated bronchial cells compared to healthy control subjects. The up-regulation of ACKR4 is linked to the disease course, as assessed by chest radiography [380]. In mice, Ackr4 also identifies a subpopulation of intestinal submucosal fibroblasts involved in endothelial cell regulation through the VEGFD/VEGFR3 and Ccl21/Ackr4 pathways [381].

ACKR5: ACKR5 (GPR182) was recently included in systematic nomenclature of chemokine receptors as a chemokine scavenger [382], binds to CCL28, CXCL10, CXCL12, and CXCL13 in high affinity with ligand overlap on CXCR3, CXCR4, and CXCR5 receptors [16,20,34,46], but also recognizes CCL1, CCL11, CCL19, CCL22, CCL24, CCL25, CCL26, CCL27, CXCL11, and CXCL14 [46]. ACKR5 is an endothelium-specific atypical chemokine receptor [47], and GPR182-deficient mice showed elevated plasma CCL22, CCL24, CCL25, CCL27, CXCL10, CXCL12, and CXCL13 levels [383]. This is correlated with significant decrease in hematopoietic stem cells in the bone marrow as well as increased colony-forming units of hematopoietic progenitors in the blood and the spleen [383]. GPR182^-/-^ mice have a reduced marginal zone, also influencing T-independent immunity [46]. Thus, ACKR5 maintains hematopoietic stem cell homeostasis, but the ACKR5’s role in IPF remains unclear.

## 5. Chemokine System in the Context of Pulmonary Fibrosis: The Transcriptional Data Similarity Between IPF and Bleomycin Model of Lung Fibrosis

Genetic and transcriptomic studies in human lung samples and murine models reveal the molecular complexity of IPF. Re-evaluating public datasets, including microarray (GSE32537 and GSE53845) and RNAseq (GSE99621) of IPF lung tissues, and microarray of bleomycin-induced lung fibrosis in mice (GSE37635) using Phantasus, for gene expression analysis (https://genome.ifmo.ru/phantasus accessed on 7 September 2023) [384], we identified up-regulated genes associated with fibrosis and the chemokine system [72,385].

The gene expression of the chemokine system in IPF is complex, as illustrated in Table 2 and Supplementary Figure S1, using public datasets from three IPF cohorts (GSE32537, GSE53845, GSE99621). Various CC and CXC chemokines (*CCL2*, *CCL5*, *CCL8*, *CCL11*, *CCL13*, *CCL19*, *CCL21*, *CCL24*, *CXCL12*, *CXCL13*, *CXCL14*) and the CC chemokine receptor *CCR7* show common up-regulation in microarray and also confirmed by RNAseq (Table 2 and Supplementary Figure S1). Some chemokines, such as *CCL3*, *CCL7*, *CCL18*, *CCL22*, *CXCL6*, *CXCL9*, *XCL2*, *CCR5*, *CCR6*, *CCR8*, *CXCR3*, *CXCR6* are up-regulated in two datasets (Table 2 and Supplementary Figure S1), while others like *CCL23*, *CXCL2*, *CXCR1*, and *ACKR4* are down-regulated, in which microarray analysis were confirmed by RNAseq (Table 2 and Supplementary Figure S1). However, we did not observe significant changes in the expression of *CCL15*, *CCL16*, *CCL17*, *CCL25*, *CCL27*, *CXCL3*, *CXCL8*, *CXCL16*, *CX3CL1*, or the receptors *CCR9*, *CCR10*, *ACKR1*, and *ACKR2* (Table 2 and Supplementary Figure S1) based on microarray and RNA sequencing analyses. In contrast, alterations in chemokine expression in lung samples from IPF patients were associated with increased expression of tissue remodeling genes linked to fibrosis, such as *COL1A2*, *COL3A1*, *COL5A2*, *COL14A1*, *COL15A1*, *COL17A1*, *MMP7*, *MMP10*, and *MMP12* (Supplementary Figure S1). These findings suggest that chemokines are closely correlated with the fibrogenesis process and may contribute to the progression of IPF.

**Figure 3:**
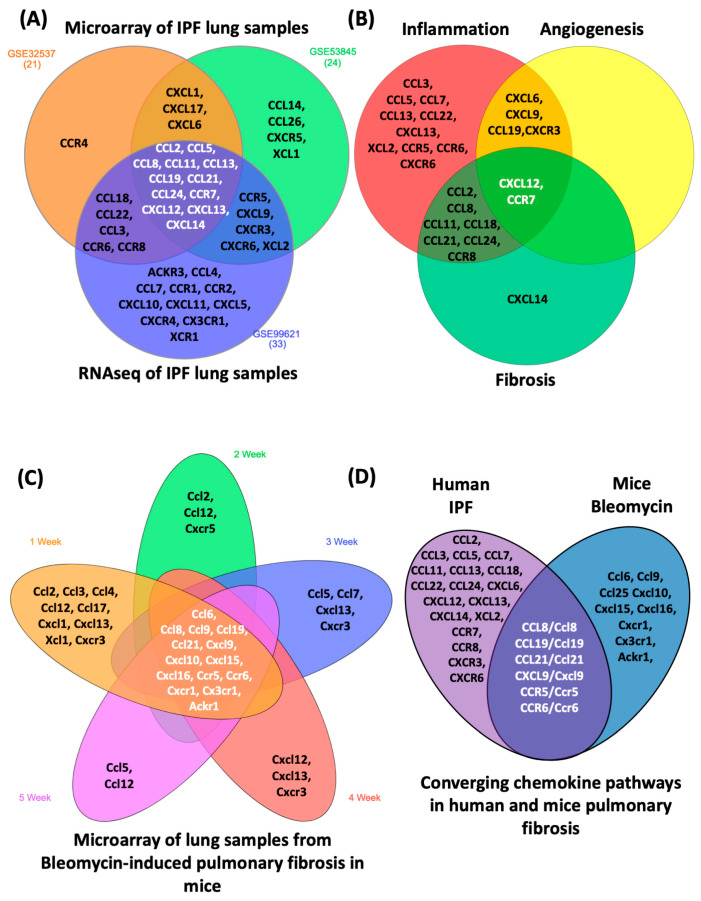
The chemokine system pathways in the context of pulmonary fibrosis: Chemokine and receptors up-regulated in IPF lung tissues by microarray analysis GEO database: (GSE32537 and GSE53845) and up-regulated in RNAseq analysis of IPF lung tissue (GEO database: GSE99621) are converging chemokine pathways in human IPF samples (**A**). Chemokine and receptors were grouped by function in IPF—Inflammation, Angiogenesis, and Fibrosis (**B**), note that some chemokines have more than one function that contributes to fibrogenesis. Chemokine and receptors up-regulated in lung tissues from bleomycin-induced pulmonary fibrosis by microarray analysis (GEO database: GSE37635) and expressed according to the developmental stages (week 1, 2, 3, 4, and 5) of pathology progression during the experimental pulmonary fibrosis in mice (**C**). Common chemokine signatures up-regulated in IPF lung tissues by microarray GEO database: GSE32537, GEO database: GSE53845, and confirmed by RNAseq analysis of IPF lung tissue (GEO database: GSE99621) and bleomycin-induced pulmonary fibrosis in mice microarray (GEO database: GSE37635) analyzed by Phantasus (**D**). There are converging chemokine pathways up-regulated in both human IPF and bleomycin-induced pulmonary fibrosis in mice by lung tissue microarray analysis and RNAseq.

Chemokines drive many other cellular mechanisms. Importantly, the chemokines expressed in IPF datasets are associated with key processes involved in fibrosis development, including chronic lung inflammation, angiogenesis, and fibrogenesis, as depicted in Figure 3A,B. Up-regulated chemokines and receptors, mainly linked to inflammatory activities (*CCL5*, *CCL7*, *CCL8*, *CCL11*, *CCL13*, *CCL18*, *CCL19*, *CCL21*, *CCL22*, *CCL24*, *CXCL6*, *CXCL10*, *CXCL12*, *CXCL13*, *CXCL14*, *CXCL17*, *CCR5*, *CCR6*, *CCR7*, and *CCR8*), that contribute to leukocyte recruitment and chronic inflammation in IPF (Figure 2 and Figure 3A,B). Some of these chemokines (*CCL19*, *CXCL6*, *CXCL12*, and *CCR7*) also directly promote pulmonary endothelial cell angiogenesis, contributing to fibrosis (Figure 2 and Figure 3A,B). Additionally, specific inflammatory chemokines (*CCL8*, *CCL11*, *CCL18*, *CCL21*, *CCL24*, *CXCL12*, *CXCL14*, *CCR7*, and *CCR8*) support fibrogenesis by recruiting and activating fibrocytes and fibroblasts in the lungs (Figure 2 and Figure 3B). Notably, the pleiotropic activities of homeostatic *CXCL12* and *CCR7* in IPF encompass chronic lung inflammation, angiogenesis, and fibrosis (Figure 2 and Figure 3B), indicating diverse chemokine production triggering critical pathways in pulmonary fibrosis.

The bleomycin-induced pulmonary fibrosis mouse model demonstrates distinct chemokine and receptor expression patterns during the inflammatory (1 week), chronic inflammation (2–3 weeks), and fibrogenic (4–5 weeks) phases (Table 2, Supplementary Figure S2 and Figure 3C). However, we did not observe significant changes in the expression of *Ccl11*, *Cxcl14*, *Cxcl17*, *Ccr7*, *Cxcr4*, *Cxcr6*, *Ackr2*, *Ackr3*, and *Ackr4* (Table 2 and Supplementary Figure S2). Initial peaking occurs in the first week with several chemokines and receptors (e.g., *Ccl2*, *Ccl3*, *Ccl4*, *Ccl6*, *Ccl8*, *Ccl9*, *Ccl12*, *Ccl17*, *Ccl19*, *Ccl21*, *Cxcl1*, Cxcl9, *Cxcl10*, *Cxcl13*, *Cxcl15*, *Cxcl16*, *Xcl1*, *Ccr5*, *Ccr6*, *Ccxr1*, *Cxcr3*, *Cx3cr1*, and *Ackr1*). Some (e.g., *Ccl6*, *Ccl8*, *Ccl9*, *Ccl19*, *Ccl21*, *Cxcl9*, *Cxcl10*, *Cxcl15*, *Cxcl16*, *Ccr5*, *Ccr6*, *Ccxr1*, *Cx3cr1*, and *Ackr1*) remain consistently up-regulated, while *Ccl25* shows down-regulation (Table 2, Supplementary Figure S2 and Figure 3C). These findings highlight key chemokines in pulmonary fibrosis development, with concurrent expression during IPF or bleomycin challenges, paralleling collagens, and MMPs’ expression implicated in fibrogenesis (Table 2, Supplementary Figures S1 and S2).

Despite differences in chemokine expression patterns between human and mouse samples, common factors emerge from these analyses, including up-regulation of chemokine genes (*CCL8*/*Ccl8*, *CCL19*/*Ccl19*, *CCL21*/*Ccl21*, *CXCL9*/*Cxcl9*) and the receptors (*CCR5*/*Ccr5*, *CCR6*/*Ccr6*) (Table 2 and Figure 3D). These factors play a crucial role in leukocyte influx, angiogenesis, and fibrosis, indicating shared chemokine pathways in inflammation, angiogenesis, and pulmonary fibrogenesis between human IPF samples and experimentally induced fibrosis in mice. Targeting these processes, particularly through GPCR and chemokine inhibition, shows promise for IPF treatment [386]. We identified that key chemokine-receptor axes offer therapeutic potential in lung fibrosis.

CCL8-CCR2 axis: The CCL8-CCR2 axis may contribute to pulmonary fibrosis. Elevated CCL8 levels in IPF patients’ BAL correlate with shorter survival, and IPF fibroblasts express CCL8, recruiting Ccr2+ macrophages and fibrocytes/fibroblasts, promoting collagen deposition [138]. This axis is a characteristic signature in lung samples from IPF and bleomycin-instilled mice (Figure 3D). Despite its association, the precise mechanism remains unknown, necessitating further studies to elucidate CCL8’s functions in human IPF. Consequently, CCL8 could be a potential pharmacological target, given the adverse effects of Carlumab (humanized anti-CCL2) administration on IPF progression [150].

CCL19-CCR7 and CCL21-CCR7 axis: CCL19 is expressed in alveolar epithelial, endothelial cells, and fibroblasts in IPF lung samples [206]. The CCL19-CCR7 axis, originating from lymphatic endothelium and expressed by CD68+ macrophages, is implicated in IPF lymphangiogenesis [187]. Fibroblasts expressing CCR7 exhibit activation and chemotactic responses to CCL21, suggesting a role in orchestrating pulmonary fibrosis development [216]. Lymphatic endothelial cells and lymphocytes in lymphoid follicles also express CCL21 [206]. CCL19 and CCL21, typically homeostatic, are induced in pulmonary samples from IPF patients and during inflammatory and fibrogenic phases in bleomycin-induced pulmonary fibrosis in mice (Figure 3D), indicating common fibrotic pathways between humans and mice. Despite their potential as critical pharmacological targets for activities on leukocytes, endothelial cells, and pulmonary fibroblasts, caution is advised due to their homeostatic nature. Further studies are needed to determine IPF therapy based on the delicate balance of the CCL19-CCR7 and CCL21-CCR7 axis, as CCR7 antagonists and specific disease indications are yet to be identified [16].

CXCL9-CXCR3 axis: CXCL9 predicts lung function decline in IPF with lower serum levels [187]. Pharmacological CXCL9 administration inhibits collagen deposition in end-stage IPF-PH patients [303], prevents TGF-β1-induced epithelial-mesenchymal cell transition in human alveolar epithelial cells in vitro [304], and induces the sIL-13Rα2 decoy receptor, suggesting its inhibitory role in fibroplasia and extracellular matrix deposition [305]. CXCL9 is up-regulated in lung samples from IPF and bleomycin-instilled mice (Figure 3D). In IPF patients, corticosteroid treatment increases CXCR3 expression in BAL lymphocytes [185], while IFNγ-1b treatment decreases CXCL9 levels [220]. Lymphocyte CXCR3 expression may contribute to chronic leukocyte influx, causing tissue damage and scarring. The CXCR3 antagonist, AMG487, inhibits leukocyte infiltration into the lungs in a bleomycin-induced inflammation model in mice [387]. Future studies are needed to clarify gaps and explore therapeutic opportunities for IPF based on the CXCL9-CXCR3 axis. Synthetic CXCR3-specific small-molecule antagonists, effective in animal models and humans, hold promise in interfering with IPF development by limiting fibroblast activation and reducing extracellular matrix production.

CCL3/CCL5-CCR5: CCR5 recognizes CCL3, CCL4, and CCL5, facilitating myeloid and lymphoid cell trafficking [10,16]. In IPF, CCL3 is detected in samples [131,152], expressed by alveolar and interstitial macrophages and fibroblasts, correlating with granulocyte influx [131]. In bleomycin-treated mice, CCL3 is produced by alveolar macrophages and bronchial epithelial cells [154,155,156,157,158]. Antibodies against CCL3 reduce lung inflammation and fibrosis [154,159]. CCL3 plays a central role in pulmonary fibrosis by controlling inflammation and fibrogenesis through leukocyte recruitment and fibrocyte migration [157]. Double-deficient mice for CCL3-CCR5 exhibit reduced pulmonary influx of TGF-β1-producing cells and less fibrosis [157]. Evasin-1, with high affinity for CCL3 [50] reduces CCL3 levels, leukocyte recruitment, and fibrosis [50,156]. Similarly, CCL5 is expressed by alveolar macrophages [162], macrophages (CD68+), and eosinophils as key sources in fibrosing alveolitis [163], with a positive correlation between CCL5 levels and eosinophils in pulmonary fibrosis [164]. In mice lungs, Ccl5 is produced after bleomycin instillation [155,156,157], associated with the influx of Ccr5+ IFNγ-producing γδ T cells, attenuating lung fibrosis in Ackr2^-/-^ mice [155]. The role of CCL4 in IPF is less understood, with elevated CCL4 in IPF BAL [131] and in mice lungs induced upon bleomycin [155,157]. Further studies are required to clarify CCR5’s role in fibrogenesis.

CCL20-CCR6 axis: CCL20 expression by epithelial cells correlates with CCR6+ lymphocyte infiltration [206] in IPF samples. Lesions in IPF exhibit increased CCR6 and IL-17 expression compared to normal lung areas [211]. IL-17A, a well-established fibrogenic inducer in murine pulmonary fibrosis models and IPF samples [388,389], activates CCR6+ Th17 lymphocytes, responding to CCL20 levels [390]. This suggests the CCL20-CCR6 axis as a crucial pharmacological target in IPF, making CCR6 inhibition an attractive direction for the future. Despite data linking CCR6 to important lung diseases, there are currently no publications describing CCR6 antagonists or neutralizing reagents for clinical use in humans [16].

## 6. The Challenge of Pharmacological Targeting the Chemokine System in Pulmonary Fibrosis

Chemokines, versatile proteins, are pivotal in diverse biological functions, regulating tissue homeostasis, responses to damage or stress, immune organization, and repair [10,16]. About 50 chemokines can interact with approximately 20 receptors in both leukocytes and non-leukocytes, influencing cell surface arrangements during inflammation and immune responses [10,16]. In treating idiopathic pulmonary fibrosis (IPF), targeting a specific chemokine or receptor interaction is preferable to minimize effects on other systems. However, translating results from animal models to human IPF has proven challenging, leading to many failed drug development efforts for lung diseases, including IPF. This prompts consideration of whether targeting multiple chemokines could be a more effective therapeutic approach. Clinical trials for lung diseases, such as COPD (CCR1 antagonist AZD4818, CXCR1/2 antagonists SCH527123/SB656933/GSK1325756B), asthma (CCR3 neutralizing monoclonal antibody ASM8, CCR3 antagonists GSK766994/DPC168/BMS-639623, CCR4 antagonist GSK2239633), pulmonary fibrosis (anti-CCL2 Carlumab), cystic fibrosis (CXCR2 antagonist SB656933), and bronchiectasis (CXCR2 antagonist AZD-5069), have explored interventions on the chemokine system. However, some trials, like those involving CCR1/AZD4818 for COPD, CCR3/GSK766994 for asthma, and CCL2/Carlumab for IPF, have shown no efficacy [16,150]. Understanding poorly studied chemokines in human IPF is crucial. Broad targeting of chemokine receptors may offer comprehensive anti-inflammatory coverage in lung diseases, but it carries the risk of modifying other systems, potentially leading to side effects like immunosuppression or secondary infections [10]. The future development of therapies targeting chemokines and their receptors necessitates a thorough understanding of the diversity and complexity of the chemokine system in IPF.

## 7. Conclusions

Chemokines crucially influence inflammation, orchestrating processes like leukocyte recruitment, angiogenesis, and fibrogenic activities in pulmonary fibrosis. Chemokines are produced by various lung cells and leukocytes (inducers), and activation of their receptors (responders) trigger diverse cellular phenomena involved in fibrogenesis (Figure 2). Shared pathways in chemokine and receptor expression between humans and mice reveal potential pharmacological targets. Identifying specific targets for disease control is challenging due to complex relationships within chemokine families and receptors. Despite potential off-target effects, side effects may be acceptable depending on disease severity. Alveolar macrophages contribute to fibrosis progression through chemokine production and regulatory functions, by chemokine source and scavenging for de-activating specific chemokines [72].

Recently, Zhao and colleagues identified and validated chemokine system-related genes associated with idiopathic pulmonary fibrosis (IPF) using public datasets [391]. Their study revealed increased expression of *CXCL2*, *XCL1*, *CCL19*, *CCL13*, *CCL11*, *CXCL6*, and *CXCL13* across two independent cohorts (GSE47460 and GSE70866), distinct from the datasets used in our analyses. Consistent with their findings, we confirmed the overexpression of *CCL11*, *CCL13*, *CCL19*, *CXCL6*, and *CXCL13*, which can be considered markers of IPF and appear central to the progression of the disease. Furthermore, our study compares the chemokine and receptor expression patterns observed in IPF with those in the bleomycin-induced pulmonary fibrosis model, emphasizing their pathway convergences. It highlights the continued relevance of this model for investigating both inflammatory and fibrogenic components of fibrosis. While the bleomycin model has faced criticism for not being a perfect representation of human IPF, our findings demonstrate that it shares significant similarities in gene expression and pathology, reinforcing its utility in fibrosis research.

In conclusion, abnormal chemokine levels in idiopathic pulmonary fibrosis (IPF) lungs activate multiple targets, underscoring the central role of chemokine signaling in fibrogenesis. The highlighted chemokine axes (CCL8-CCR2, CCL19/CCL21-CCR7, CXCL9-CXCR3, CCL3/CCL5-CCR5, and CCL20-CCR6) offer promising research avenues and potential pharmacological targets for IPF treatment. Fine-tuning chemokine levels could reverse pulmonary fibrotic manifestations, and further studies and clinical trials will validate this approach, potentially providing new therapeutic opportunities for IPF management.

## Figures and Tables

**Table 1:** Complexity of chemokine system: Chemokines and their receptors. The complexity of the chemokine system and its functions are illustrated based on reviewed literature. Blue correspond to the chemokines that naturally antagonizes the chemokine receptors.

Chemokine System		
**Chemokines**	**Other Name (s)**	**Chemokine Receptors**
CCL1	I-309, TCA-3	CCR8, ACKR1
CCL2	MCP-1, JE	CCR2, ACKR1, ACKR2, ACKR5
CCL3	MIP-1α	CCR1, CCR5, ACKR2
CCL4	MIP-1β	CCR1, CCR3, CCR5, ACKR2
CCL5	RANTES	CCR1, CCR2, CCR3, CCR5, ACKR1, ACKR2
CCL6	C10, MRP-2	CCR1, ACKR2
CCL7	MARC, MCP-3	CCR1, CCR2, CCR3, CCR5, ACKR1, ACKR2
CCL8	MCP-2	CCR1, CCR2, ACKR1, ACKR2
CCL9/CCL10	MRP-2, CCF18, MIP-1γ	CCR1
CCL11	Eotaxin	CCR2, CCR3, CCR5, ACKR1, ACKR2
CCL12	MCP-5	CCR2, ACKR2
CCL13	MCP-4, NCC-1	CCR1, CCR2, CCR3, ACKR1, ACKR2
CCL14	HCC-1, MCIF, NCC-2	CCR1, CCR5, ACKR1, ACKR2
CCL15	Leukotactin-1, MIP-5, HCC-2, NCC-3	CCR1, CCR3
CCL16	LEC, NCC-4, LMC,	CCR1, CCR2, CCR5, CCR8, ACKR1, ACKR5
CCL17	TARC, ABCD-2	CCR4, ACKR1, ACKR2, ACKR5
CCL18	PARC, DC-CK1, AMAC-1, MIP-4	CCR8, ACKR1
CCL19	ELC, Exodus-3	CCR7, ACKR4, ACKR5
CCL20	LARC, Exodus-1	CCR6, ACKR4, ACKR5
CCL21	SLC, 6Ckine, Exodus-2, TCA-4	CCR7, ACKR4, ACKR5
CCL22	MDC	CCR4, ACKR2, ACKR4, ACKR5
CCL23	MPIF-1, MIP-3, MPIF-1	CCR1, ACKR2
CCL24	Eotaxin-2, MPIF-2,	CCR3, ACKR2
CCL25	TECK	CCR9, ACKR4
CCL26	Eotaxin-3, MIP-4α, IMAC, TSC-1	CCR1, CCR2, CCR3, CCR5, ACKR2
CCL27	CTACK, ILC	CCR10
CCL28	MEC	CCR3, CCR10, ACKR5
CXCL1	Gro-α, GRO1, NAP-3, KC	CXCR2, ACKR1
CXCL2	Gro-β, GRO2, MIP-2α	CXCR2, ACKR1, ACKR2
CXCL3	Gro-γ, GRO3, MIP-2β	CXCR2, ACKR1
CXCL4	PF-4	CXCR3, ACKR1, ACKR5
CXCL5	ENA-78	CXCR1, CXCR2, ACKR1
CXCL6	GCP-2	CXCR1, CXCR2, ACKR1
CXCL7	NAP-2, CTAPIII, PEP	CXCR2
CXCL8	IL-8, NAP-1, MDNCF, GCP-1	CXCR1, CXCR2, ACKR1
CXCL9	MIG, CRG-10	CXCR3, CCR3, ACKR1, ACKR5
CXCL10	IP-10, CRG-2	CXCR3, CCR3, ACKR1, ACKR2, ACKR5
CXCL11	I-TAC, IP-9	CXCR3, CCR3, CCR5, ACKR1, ACKR3, ACKR5
CXCL12	SDF-1, PBSF	CXCR4, ACKR1, ACKR3, ACKR5
CXCL13	BCA-1, BLC	CXCR5, ACKR1, ACKR4, ACKR5
CXCL14	BRAK	Unknown, ACKR5
CXCL15	Lungkine, WECHE	Unknown
CXCL16	SRPSOX	CXCR6
CXCL17	DMC, VCC-1	CXCR8
XCL1	Lymphotactin α	XCR1
XCL2	Lymphotactin β	XCR1
CX3CL1	Fractalkine, Neurotactin	CX3CR1
**Atypical Chemokine Receptors**	**Other name (s)**	**Chemokines**
ACKR1	DARC, CD234, Duffy antigen	CCL1, CCL2, CCL5, CCL7, CCL8, CCL11, CCL12 CCL13, CCL14, CCL16, CCL17, CCL18, CXCL1, CXCL2, CXCL3, CXCL4, CXCL5, CXCL6, CXCL8, CXCL9, CXCL10, CXCL11, CXCL12, CXCL13
ACKR2	D6, CCBP2, CMKBR9	CCL2, CCL3, CCL3L1, CCL4, CCL5, CCL6, CCL7, CCL8, CCL11, CCL12, CCL13, CCL14, CCL17, CCL22, CCL23, CCL24, CCL26, CXCL2, CXCL10, CXCL14
ACKR3	CXCR7, RDC1, CMKOR1	CXCL11, CXCL12, vCCL2
ACKR4	CCX-CKR, CCRL1, CCR11	CCL19, CCL20, CCL21, CCL22, CCL25, CXCL13
ACKR5	GPR182	CCL2, CCL16, CCL17, CCL19, CCL20, CCL21, CCL22, CCL28, CXCL4, CXCL9, CXCL10, CXCL11, CXCL12, CXCL13, CXCL14

References: [10,16,19,20].

**Table 2:** Summary of the chemokine system transcriptional analysis in the context of IPF: Microarray analysis of IPF patient lung tissues, GEO database: GSE32537, GEO database: GSE53845, and RNAseq analysis of lung tissue from IPF GEO database: GSE99621 and analysis of lung samples from bleomycin-induced lung fibrosis in C57BL/6 mice GEO database: GSE37635 by *Phantasus*. The symbols correspond to (+) up-regulated, (−) down-regulated, (=) no changes related to the controls, and (n.a.) not analyzed in respective datasets. Red corresponds with (+) up-regulated found in three human IPF datasets (GSE32537, GSE53845, GSE99621), or five time-points after bleomycin in mice (GSE37635). Purple corresponds with (+) up-regulated or (−) down-regulated, (=) no changes related to the controls found at list one microarray (GSE32537 or GSE53845) and confirmed by RNAseq (GSE99621) human IPF datasets, or found at list four time-points after bleomycin in mice. Blue corresponds to the genes with no changes related to the controls (=), found at list one microarray (GSE32537 or GSE53845) and confirmed by RNAseq (GSE99621) human IPF datasets, or five time-points after bleomycin in mice (GSE37635).

Tissue	Biopsy of Lung Tissue from IPF Patients			Lung Tissue from Bleomycin-Induced Pulmonary Fibrosis in Mice					
	Microarray		RNAseq	Microarray					
GEO	GSE32537	GSE53845	GSE99621	GSE37635					
					*1w*	*2w*	*3w*	*4w*	*5w*
Gene				Gene					
CC chemokines									
*CCL1*	n.a.	=	+						
*CCL2*	+	+	+	*Ccl2*	+	+	=	=	=
*CCL3*	+	=	+	*Ccl3*	+	=	=	=	=
*CCL4*	=	=	+	*Ccl4*	+	=	=	=	=
*CCL5*	+	+	+	*Ccl5*	=	=	+	=	+
				*Ccl6*	+	+	+	+	=
*CCL7*	+	=	+	*Ccl7*	=	=	+	=	=
*CCL8*	+	+	+	*Ccl8*	+	+	+	+	+
				*Ccl9*	+	+	+	+	=
*CCL11*	+	+	+	*Ccl11*	=	=	=	=	=
				*Ccl12*	+	+	=	=	+
*CCL13*	+	+	+						
*CCL14*	n.a.	+	=						
*CCL15*	=	=	=						
*CCL16*	n.a.	=	=						
*CCL17*	n.a.	=	=	*Ccl17*	+	=	=	=	=
*CCL18*	+	=	+						
*CCL19*	+	+	+	*Ccl19*	+	+	+	+	=
*CCL20*	−	=	=						
*CCL21*	+	+	+	*Ccl21*	=	+	+	+	+
*CCL22*	+	=	+						
*CCL23*	−	−	=						
*CCL24*	+	+	+						
*CCL25*	n.a.	=	=	*Ccl25*	=	−	−	−	−
*CCL26*	n.a.	+	−						
*CCL27*	n.a.	=	=	*Ccl27*	−	=	=	=	=
*CCL28*	−	=	−						
CXC chemokines									
*CXCL1*	+	+	=	*Cxcl1*	+	=	=	=	=
*CXCL2*	−	=	=						
*CXCL3*	=	=	=						
*CXCL4*	n.a.	−	=	*Cxcl4*	=	−	=	=	=
*CXCL5*	=	=	+						
*CXCL6*	+	−	+						
*CXCL7*	n.a.	n.a.	n.a.						
*CXCL8*	=	=	=						
*CXCL9*	=	+	+	*Cxcl9*	+	+	+	+	=
*CXCL10*	=	=	+	*Cxcl10*	+	+	+	+	=
*CXCL11*	=	=	+						
*CXCL12*	+	+	+	*Cxcl12*	=	=	=	+	=
*CXCL13*	+	+	+	*Cxcl13*	+	=	+	+	=
*CXCL14*	+	+	+	*Cxcl14*	=	=	=	=	=
				*Cxcl15*	+	+	+	+	+
*CXCL16*	=	=	=	*Cxcl16*	+	+	+	+	=
*CXCL17*	+	+	=	*Cxcl17*	=	=	=	=	=
CX3C chemokine									
*CX3CL1*	=	=	=	*Cx3cl1*	−	=	=	=	=
XC chemokines									
*XCL1*	=	+	=	*Xcl1*	+	=	=	=	=
*XCL2*	n.a.	+	+						
CC chemokine receptors									
*CCR1*	−	=	+						
*CCR2*	=	=	+						
*CCR3*	n.a.	−	=						
*CCR4*	+	=	=						
*CCR5*	=	+	+	*Ccr5*	+	+	+	+	+
*CCR6*	+	−	+	*Ccr6*	+	+	+	+	=
*CCR7*	+	+	+	*Ccr7*	=	=	=	=	=
*CCR8*	+	=	+						
*CCR9*	n.a.	=	=						
*CCR10*	n.a.	=	=						
CXC chemokine receptors									
*CXCR1*	−	−	=	*Cxcr1*	+	+	+	+	=
*CXCR2*	−	−	=						
*CXCR3*	n.a.	+	+	*Cxcr3*	+	=	+	+	=
*CXCR4*	=	=	+	*Cxcr4*	=	=	=	=	=
*CXCR5*	=	+	=	*Cxcr5*	=	+	=	=	=
*CXCR6*	=	+	+	*Cxcr6*	=	=	=	=	=
CX3C chemokine receptor									
*CX3CR1*	=	−	+	*Cx3cr1*	+	+	+	+	+
XC chemokine receptor									
*XCR1*	=	=	+						
Atypical chemokine receptors									
*ACKR1*	=	=	=	*Ackr1*	+	+	+	=	+
*ACKR2*	n.a.	=	=	*Ackr2*	=	=	=	=	=
*ACKR3*	n.a.	=	+	*Ackr3*	=	=	=	=	=
*ACKR4*	−	−	=	*Ackr4*	=	=	=	=	=

## Data Availability

The datasets presented in this study are public-deposited data and can be found in online repositories. The name of the repository and accession numbers can be found below: https://www.ncbi.nlm.nih.gov/geo/ accessed on 9 July 2023, microarray (GSE32537 and GSE53845) and RNAseq (GSE99621) of IPF lung tissues, and microarray of bleomycin-induced lung fibrosis in mice (GSE37635).

## Supplementary Materials

The following supporting information can be downloaded at: https://www.mdpi.com/article/10.3390/cells13242058/s1, Figure S1: The complexity of the chemokine system in the context of Idiopathic Pulmonary Fibrosis of human samples; Figure S2: The complexity of the chemokine system in the context of Bleomycin-induced pulmonary fibrosis of mice samples.
