# Supplementary figures and images for: The Chemokine System as a Key Regulator of Pulmonary Fibrosis: Converging Pathways in Human Idiopathic Pulmonary Fibrosis (IPF) and the Bleomycin-Induced Lung Fibrosis Model in Mice
Source: Cells. 2024 Dec 12;13(24):2058. doi: 10.3390/cells13242058 (PMC11674266; doi:10.3390/cells13242058)

Supplementary figures:  
Supplementary figure S1:

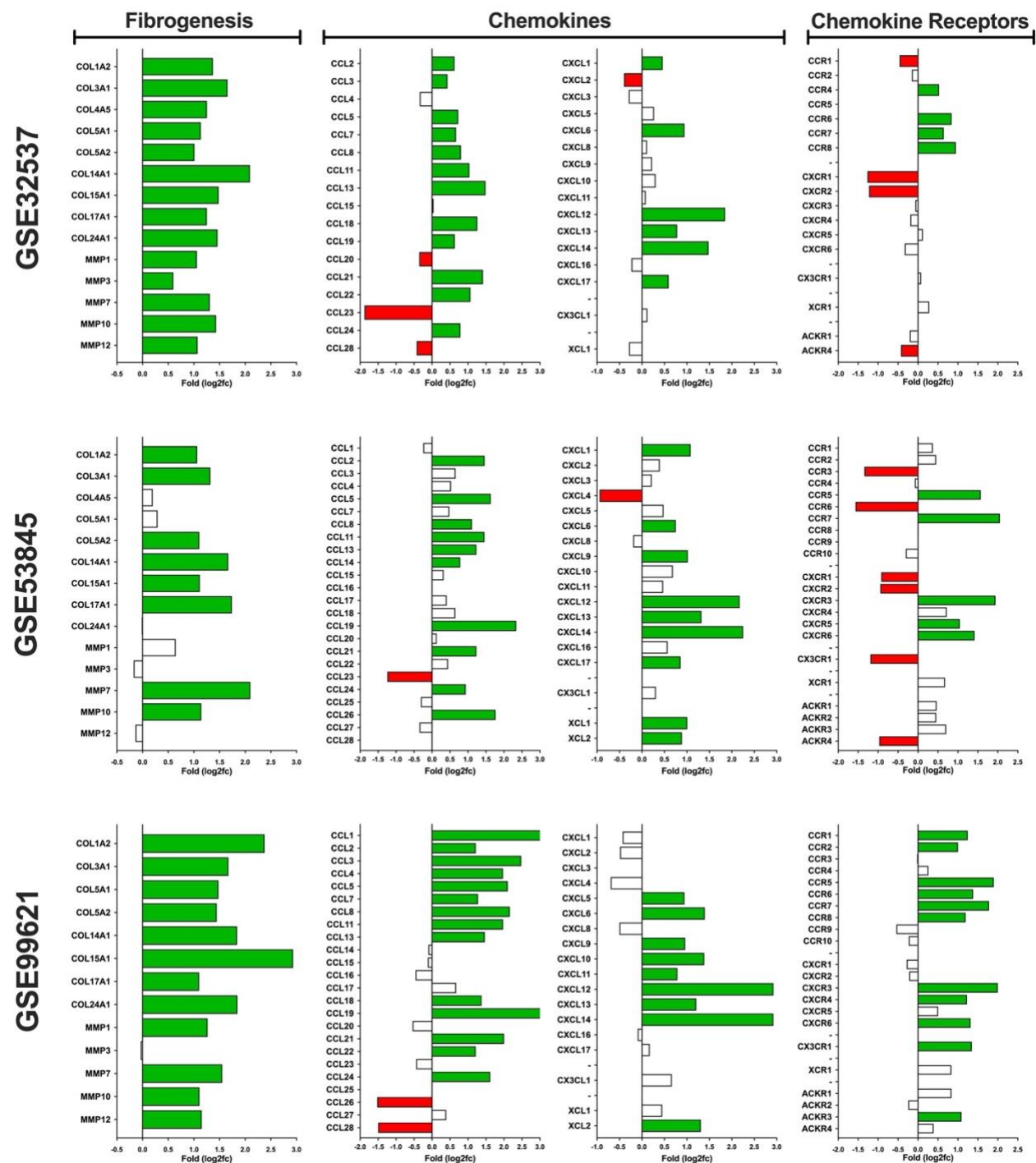

Supplementary figure S2:

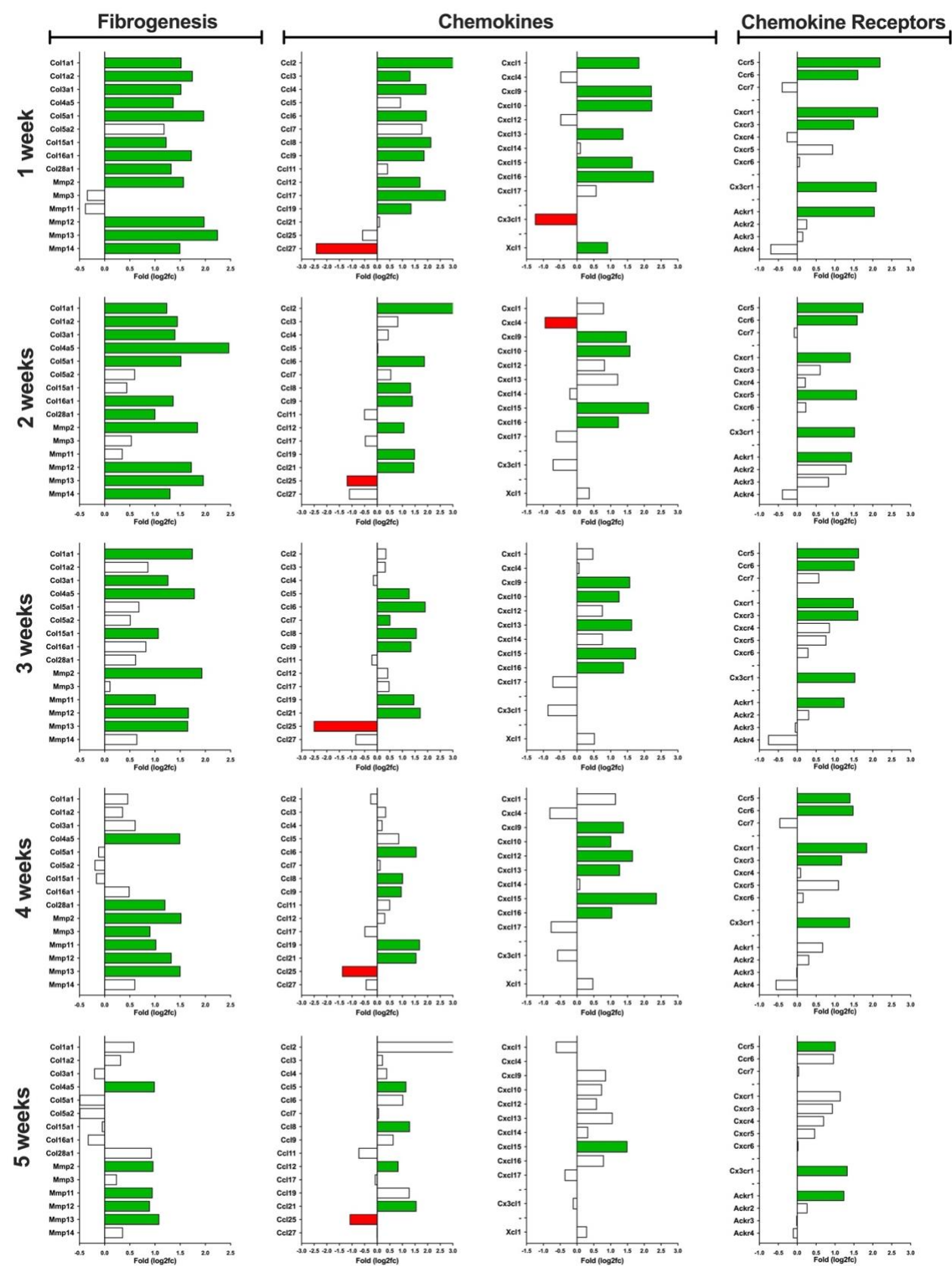

Supplement: Supplementary file 1 [file cells-13-02058-s001.zip › cells-3327504-supplementary.pdf]
